# Homologous-magnetic dual-targeted metal-organic framework to improve the Anti-hepatocellular carcinoma efficacy of PD-1 inhibitor

**DOI:** 10.1186/s12951-024-02469-6

**Published:** 2024-04-24

**Authors:** Hong Guo, Xia Li, Dengxuan Mao, Hong Wang, Liangyin Wei, Ding Qu, Xiaoying Qin, Xiaoqi Li, Yuping Liu, Yan Chen

**Affiliations:** 1 Affiliated Hospital of Integrated Traditional Chinese and Western Medicine, Nanjing University of Chinese Medicine, 100 Hongshan Rd, Qixia Qu, Nanjing, Jiangsu 210028 China; 2 Multi-component of Traditional Chinese Medicine and Microecology Researh Center, Jiangsu Probince Academy of Traditional Chinese Medicine, Nanjing, Jiangsu 210028 China; 3 Jiangsu Clinical Innovation Center of Digestive Cancer of Traditional Chinese Medicine, Nanjing, Jiangsu 210028 China

**Keywords:** PD-1 inhibitor, Tumour-infiltrating T lymphocytes, Homologous–magnetic dual-targeting, Tumour blood vessels, Combination therapy

## Abstract

The insufficient abundance and weak activity of tumour-infiltrating lymphocytes (TILs) are two important reasons for the poor efficacy of PD-1 inhibitors in hepatocellular carcinoma (HCC) treatment. The combined administration of tanshinone II_A_ (TSA) and astragaloside IV (As) can up-regulate the abundance and activity of TILs by normalising tumour blood vessels and reducing the levels of immunosuppressive factors respectively. For enhancing the efficacy of PD-1 antibody, a magnetic metal–organic framework (MOF) with a homologous tumour cell membrane (Hm) coating (Hm@TSA/As-MOF) is established to co-deliver TSA&As into the HCC microenvironment. Hm@TSA/As-MOF is a spherical nanoparticle and has a high total drug-loading capacity of 16.13 wt%. The Hm coating and magnetic responsiveness of Hm@TSA/As-MOF provide a homologous-magnetic dual-targeting, which enable Hm@TSA/As-MOF to counteract the interference posed by ascites tumour cells and enhance the precision of targeting solid tumours. Hm coating also enable Hm@TSA/As-MOF to evade immune clearance by macrophages. The release of TSA&As from Hm@TSA/As-MOF can be accelerated by HCC microenvironment, thereby up-regulating the abundance and activity of TILs to synergistic PD-1 antibody against HCC. This study presents a nanoplatform to improve the efficacy of PD-1 inhibitors in HCC, providing a novel approach for anti-tumour immunotherapy in clinical practice.

**Supplementary Information:**

The online version contains supplementary material available at 10.1186/s12951-024-02469-6.

## Introduction

Hepatocellular carcinoma (HCC) is the third leading cause of cancer-related deaths worldwide, ranking sixth in terms of incidence [1]. Existing treatment approaches for HCC mainly include surgery, radiotherapy and other modalities. These approaches often yield limited benefits for patients and have apparent side effects [2–4]. In recent years, immunotherapies, particularly anti-PD-1 therapy, have demonstrated promising efficacy in early-phase trials [5, 6]. However, in clinical settings, the objective response rate of PD-1 inhibitors as monotherapy is relatively low, ranging from 15 to 20%, in patients with HCC [7]. Consequently, developing strategies for enhancing the therapeutic efficacy of PD-1 inhibitors is necessary.

PD-1 inhibitors exert anti-tumour effects by restoring the immune response of tumour-infiltrating lymphocytes (TILs) [8]. Therefore, the abundance and activity of TILs directly determine the therapeutic efficacy of PD-1 inhibitors in HCC [9]. In the HCC microenvironment, vessels exhibit pronounced hyperplasia, severe ruptures and hypoperfusion, which hinder the intra-tumoral infiltration of TILs [10–12]. Tanshinone II_A_ (TSA), a diterpenoid compound from Salviae miltiorrhizae radix et rhizoma, has been demonstrated to inhibit the secretion of VEGF and regulate vascular normalisation [13–16]. Therefore, tumour vessel normalisation through TSA has the potential to increase the abundance of TILs. However, TILs still exhibit insufficient activity. This limitation is attributed to the increased levels of immunosuppressive factors such as TGF-β1 within HCC tissues, which inhibit the activity of TILs [17]. Astragaloside IV (As), a tetracyclic triterpenoid compound from Astragali radix, has been shown to inhibit the secretion of TGF-β1 [18] and enhance the activity of TILs [19, 20]. We hypothesised that combination therapy with TSA and As might increase the abundance and activity of TILs simultaneously, thereby enhancing the therapeutic efficacy of PD-1 inhibitors in HCC. Therefore, achieving the co-delivery of TSA and As to the HCC microenvironment is key to improving the anti-HCC effects of PD-1 inhibitors.

However, both TSA and As have poor water solubility, which leads to low bioavailability and poor biodistribution [21–24]. Using nanosystems for delivering drugs to target lesions is an efficient strategy for enhancing the bioavailability and biodistribution of drugs. Conventional nanosystems such as microemulsion and micelle are generally designed for single-drug delivery and frequently have a low drug-loading capacity (DLC). Therefore, effectively co-delivering TSA and As to the HCC microenvironment is challenging. Metal-organic frameworks (MOFs) are promising organic-inorganic porous materials that are constructed with metal nodes and organic linkers [25–27]. Their highly adaptive pores environment and large surface area enable the co-loading of multiple drugs and offer a high DLC [28], suggesting that MOFs are an ideal carrier for the co-loading of TSA and As.

The capture and clearance of nanoparticles by the mononuclear phagocyte system (MPS) can reduce the therapeutic efficacy of drug. An effective solution to address this issue is using endogenous cell membranes as camouflage coatings to create a biomimetic nanoplatform [18]. Amongst various cell membranes, homologous tumour cell membranes offer a dual advantage by deceiving the MPS to evade clearance while concurrently improving the biodistribution of TSA and As through homologous targeting [27, 29, 30]. However, a notable challenge arises from the abundance of homologous tumour cells in ascites, a typical clinical feature of HCC, which substantially impedes the precise targeting capability of tumour cell membranes coating toward solid tumours. To address this issue, we designed a homologous-magnetic dual-targeted nanoplatform based on a magnetic MOF constructed using Fe_3_O_4_ as the metal node [31]. The homologous-magnetic nanoplatform selectively targeted solid tumours of HCC instead of ascites owing to its responsiveness to magnetic fields.

**Scheme 1:**
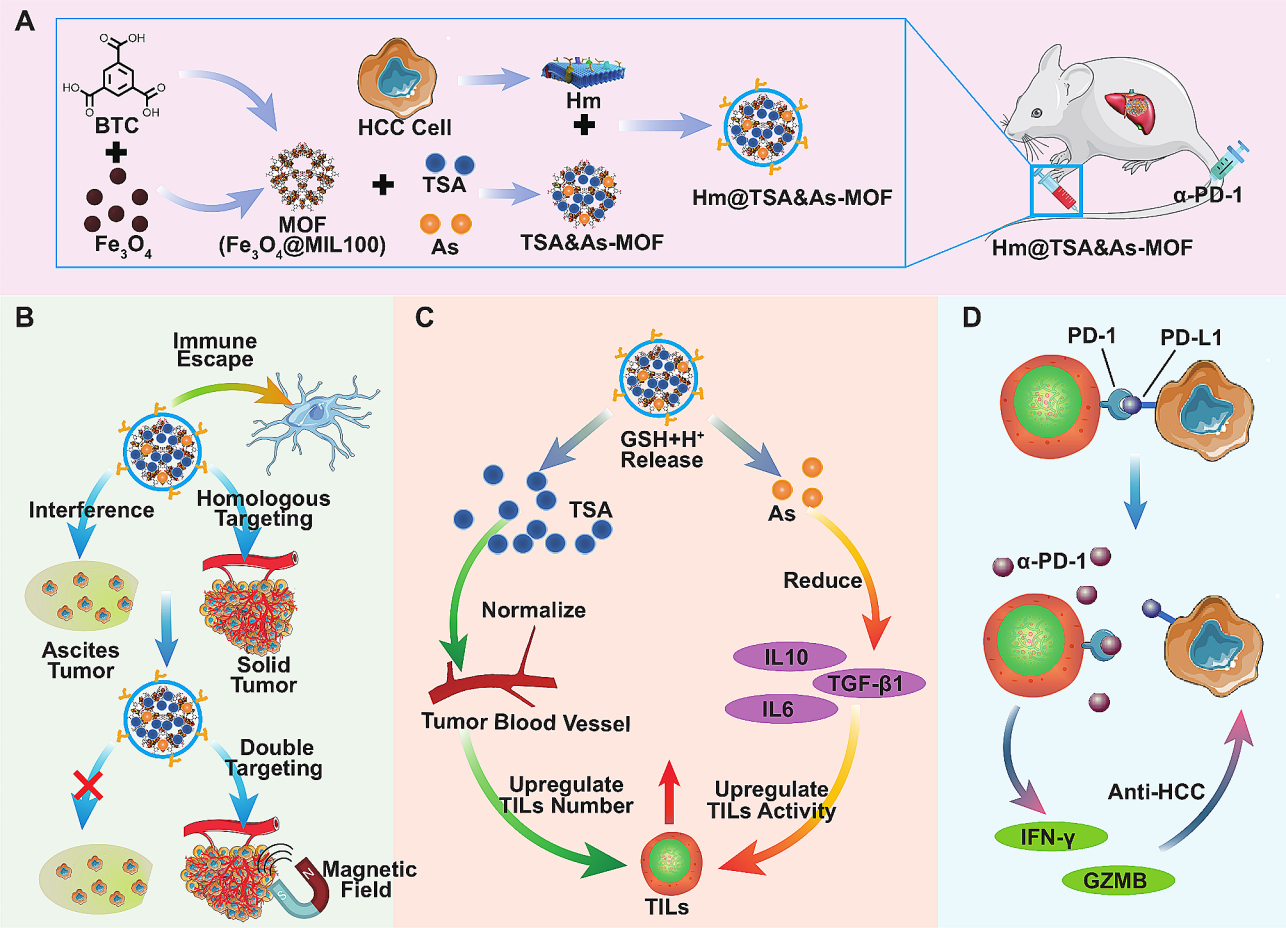
Schematic demonstration of combination therapy of Hm@TSA/As-MOF and α-PD-1 in HCC. (**A**) MOFs were synthesised using Fe_3_O_4_ nanoparticles as precursors, followed by post-loading to form TSA/As-MOFs. Subsequently, homologous tumour cell membranes (Hm) were coated on the surface of TSA/As-MOFs to obtain Hm@TSA/As-MOFs. Finally, Hm@TSA/As-MOFs were injected into mice with HCC through the tail vein. (**B**) Hm@TSA/As-MOFs evaded clearance by the MPS owing to the Hm. Under a magnetic field, Hm@TSA/As-MOFs counteracted the interference of ascites tumour cells on Hm, resulting in more accurate targeting of solid tumours. (**C**) In the reductive and acidic microenvironment of HCC, TSA and As were rapidly released from Hm@TSA/As-MOFs. TSA increased the abundance of TILs by normalising tumour blood vessels, and As upregulated the activity of TILs by reducing the levels of immunosuppressive factors. (**D**) The combined application of Hm@TSA/As-MOFs and α-PD-1 enhanced the overall anti-tumour effects

Herein, we present a homologous-magnetic dual-targeted nanoplatform, named Hm@TSA/As-MOF, to enhance the therapeutic effects of PD-1 antibody (α-PD-1) against HCC. This nanoplatform is composed of magnetic MOFs and coated with homologous tumour cell membranes for the co-delivery of TSA and As into the HCC microenvironment. It can evade clearance by MPS owing to the presence of tumour cell membranes and precisely deliver TSA and As to HCC tissues *via* homologous-magnetic dual-targeting. The coordination bonds within the structure of Hm@TSA/As-MOF have low stability in acidic and reducing environments. This characteristic enables Hm@TSA/As-MOF to respond to the HCC microenvironment, triggering the release of TSA and As. Furthermore, the nanoplatform can simultaneously increase the abundance of TILs by promoting tumour blood vessel normalisation and improve the activity of TILs by reducing the levels of immunosuppressive factors. Ultimately, Hm@TSA/As-MOF is used to synergize with α-PD-1 to enhance the anti-HCC effect (Scheme 1). This strategy represents a sustained and efficient drug delivery system with promising potential in the immunotherapy of HCC in clinical settings.

## Results and discussions

### Fabrication and characterisation of Hm@TSA/As-MOF

Fe_3_O_4_ nanoparticles were synthesized using a solvothermal method described previously [32]. A magnetic MOF, Fe_3_O_4_@MIL100, was synthesised using Fe_3_O_4_ nanoparticles as precursors [33]. Subsequently, the magnetic MOF was immersed in a methanol solution containing TSA and As for 12 h to obtain TSA/As-MOF. Finally, the homologous H22 cell membrane was coated on the surface of TSA/As-MOF through sonication, resulting in the formation of biomimetic–magnetic Hm@TSA/As-MOF [34, 35]. 

**Fig. 1:**
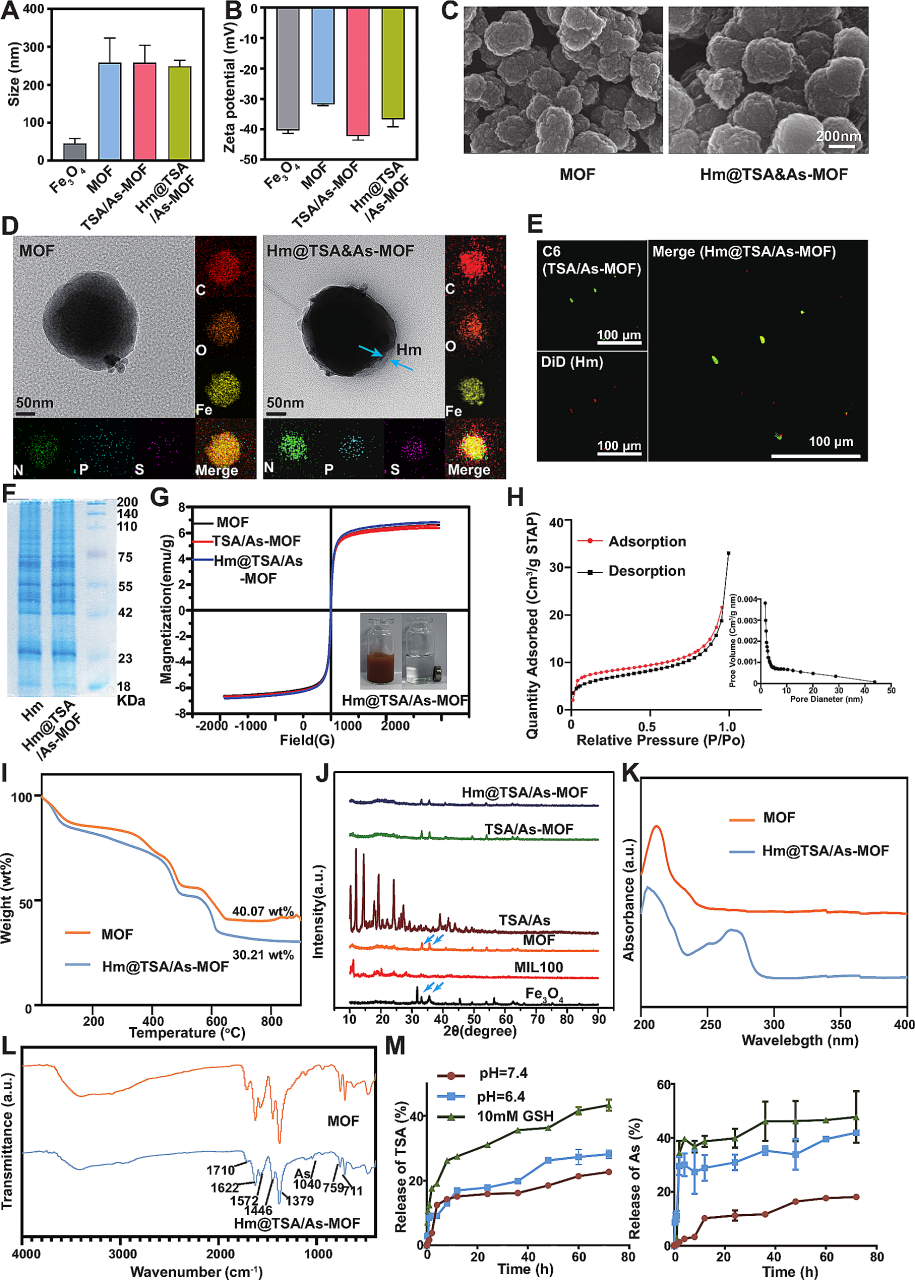
Characterisation of Hm@TSA/As-MOF. The particle size distribution (**A**) and zeta potential (**B**) of Fe_3_O_4_, MOF, TSA/As-MOF and Hm@TSA/As-MOF were determined via DLS (*n* = 3). Representative images of SEM (**C**) (scale bar = 200 nm) and TEM (**D**) (scale bar = 50 nm) of MOF and Hm@TSA/As-MOF are shown. The co-localization of Hm and TSA/As-MOF in Hm@TSA/As-MOF was demonstrated by laser confocal microscopy (**E**). The protein profile of Hm@TSA/As-MOF was examined via SDS-PAGE (**F**). The responsiveness of MOF, TSA/As-MOF and Hm@TSA/As-MOF to magnetic fields was tested using VSM (**G**). The pore characteristics of Hm@TSA/As-MOF was observed by the curves of nitrogen adsorption and desorption (**H**). The TGA was tested in a N_2_ environment (**I**). The crystal structure of Fe_3_O_4_, MIL-100 (Fe), MOF, TSA/As, TSA/As-MOF and Hm@TSA/As-MOF was examined via XRD (**J**). The UV (**K**) and FTIR (**L**) spectras of TSA/As-MOF and Hm@TSA/As-MOF are shown. The release profiles of TSA and As at different conditions (pH 7.4, pH 6.4 and 10-mM GSH) (*n* = 3) (**M**) are shown. Data are expressed as the mean ± SD

Dynamic light scattering (DLS) was employed to assess the size and zeta potential of nanoparticles. Initially, the size of Fe_3_O_4_ nanoparticles was 53.24 ± 4.13 nm. Upon synthesis into the MOF, the size increased to 206.50 ± 51.90 nm. The size of TSA/As-MOF reached 258.3 ± 25.2 nm, whereas that of final Hm@TSA/As-MOF was 248.60 ± 16.20 nm (Fig. 1A). The zeta potential of Fe_3_O_4_ nanoparticles was -50.5 ± 1.06 mV, whereas that of MOF, TSA/As-MOF and Hm@TSA/As-MOF was -34.57 ± 0.85 mV, -42.30 ± 1.27 mV and -35.90 ± 0.70 mV, respectively (Fig. 1B). These changes in size and zeta potential suggested the successful encapsulation of drugs and coating of Hm on nanoparticles. In addition, the results of DLS showed that the size and zeta potential of Hm@TSA/As-MOF did not change significantly after its incubation with PBS (pH 7.4) for 7 days (Figure S1A), suggesting that Hm@TSA/As-MOF was experimentally stable.

Scanning electron microscopy (SEM) and transmission electron microscopy (TEM) images (Fig. 1 C, D; Figure S1A) revealed that Hm@TSA/As-MOF exhibited spherical morphology and had a Hm coating on the surface, which was similar to the previous reports [36, 37]. Furthermore, energy-dispersive spectrometry (EDS) was used to analyse the elemental distribution of Hm@TSA/As-MOF. The results showed that MOF was primarily composed of elements such as C, O and Fe, whereas Hm@TSA/As-MOF additionally contained elements such as N, P and S, originating from the proteins and phospholipids of the H22 cell membrane (Fig. 1D; Figure S1C).

The magnetic responsiveness and Hm coating of Hm@TSA/As-MOF can enable homologous-magnetic dual targeting, which promotes precise drug delivery. Laser confocal microscope images of Hm@TSA/As-MOF (Fig. 1E; Figure S1D) showed an obvious co-localization of the fluorescence signals of Hm and TSA/As-MOF, which supported the results of TEM. The membrane protein components of Hm@TSA/As-MOF were analysed via SDS-PAGE (Fig. 1F). The results verified that Hm coating did not cause significant protein loss, which was essential for the homologous targeting and immune evasion capabilities of Hm@TSA/As-MOF. As analysed using a vibrating-sample magnetometer (VSM), the magnetisation value of Hm@TSA/As-MOF was estimated to be 6 emu▪g^-1^, and adsorption analysis by a NdFeB permanent magnet further demonstrated the magnetic responsiveness of Hm@TSA/As-MOF (Fig. 1G; Figure S1E, F).

In conventional nanocarriers, such as microemulsions and mesoporous silica, loading more than 2 wt% of TSA is often challenging [21, 38], thereby constraining its pharmacological effectiveness. Moreover, conventional nanocarriers usually require a high degree of agreement with the properties of drugs to facilitate successful loading. Conventional nanocarriers, such as microemulsions, require that drugs have a good solubility in the internal phase to successfully load drugs. However, TSA and As have different solubility properties, making it difficult to co-load them in this kind of nanocarriers. Herein, Hm@TSA/As-MOF exhibited a high DLC for TSA (12.83 wt%) and enabled the concurrent loading of As (3.30 wt%) at an optimised drug mass ratio of approximately 4:1, which is consistent with the findings of our previous study [21]. Hm@TSA/As-MOF exhibited a high total DLC of 16.13 wt%, and the high DLC might be attributed to the high surface area and pore volume of MOF: before drug loading, the BET (Brunauer-Emmett-Teller) surface area of MOF was 832.31 m²▪g^-1^, both BJH (Barret-Joyner-Halenda) adsorption and desorption pore volume of MOF were 0.27 cm³▪g^-1^ [37]; after drug loading, the BET surface area of Hm@TSA/As-MOF was 22.15 m²▪g^-1^, BJH adsorption and desorption pore volume were 0.05 cm³▪g^-1^ and 0.04 cm³▪g^-1^, respectively (Fig. 1H; Figure S1G).

The thermogravimetric analysis (TGA) was tested in a N_2_ environment (Fig. 1I). MOF showed a 59.93 wt% weight loss and Hm@TSA/As-MOF showed a 69.79 wt% weight loss at 900 ℃. The additional weight loss (9.86 wt%) of Hm@TSA/As-MOF might due to the decomposition of Hm, TSA and As (Figure S1H). X-ray diffraction (XRD) was used to analyse the crystal structure of Hm@TSA/As-MOF. The XRD pattern of Hm@TSA/As-MOF revealed six distinct diffraction peaks (2θ: 30.2, 33.0, 35.6, 43.3, 53.7, 57.3 and 62.8°) and exhibited good agreement with the XRD patterns of MIL-100 (Fe) and Fe_3_O_4_ (2θ: 33.0, 35.6) nanoparticles (Fig. 1J). It did not show characteristic peaks of TSA and As, indicating that the two components were present in an amorphous form within Hm@TSA/As-MOF. The characteristic peaks of TSA/As-MOF and Hm@TSA/As-MOF were found to be consistent, suggesting that Hm coating did not alter the crystal structure of the Hm@TSA/As-MOF (Fig. 1I).

In the UV spectra of Hm@TSA/As-MOF, the peak at around 270 nm was the characteristic peak of TSA, and it was not observed to have a significantly redshift (Fig. 1K; Figure. S1I), which suggested that there was no significant conjugation between TSA and MOF. In the FTIR spectra of Hm@TSA/As-MOF, characteristic peaks at 711, 759 cm^-1^ were attributed to the C-H of the aromatic ring; characteristic peaks at 1379, 1446 cm^-1^ were attributed to the COO- of the aromatic ring; characteristic peaks at 1572, 1622, 1710 cm^-1^ were attributed to the C = O of the carboxyl (Fig. 1L; Figure S1J) [39]. Unlike MOF, Hm@TSA/As-MOF had a small peak at 1040 cm^-1^, which was the characteristic peak of As. Compared to the As, the content of As in Hm@TSA/As-MOF (only 3.30 wt%) was lower, therefore, the response value of this peak was low (Fig. 1L; Figure S1J).

The release of TSA and As from Hm@TSA/As-MOF was detected in different environments. The results showed that Hm@TSA/As-MOF released 18.69% of TSA and 8.13% of As in an environment with a pH of 7.4 (physiological), 34.83% of TSA and 35.56% of As in an environment with a pH of 6.4 (acidic) and 43.31% of TSA and 43.43% of As in an environment with 10-mM GSH (reductive) after 72 h of incubation. These results indicated that Hm@TSA/As-MOF could respond to the HCC microenvironment and enable rapid drug release (Fig. 1M).

Altogether, we successfully prepared a magnetic MOF with homologous H22 membrane coating to co-load TSA and As. Hm@TSA/As-MOF exhibited a good magnetic response and enabled rapid release of TSA and As in response to an HCC microenvironment, thereby enhancing drug efficacy.

### Cellular uptake of Hm@TSA/As-MOF

To examine the internalisation of Hm@TSA/As-MOF by vascular endothelial cells, TILs and HCC cells, we selected coumarin 6 (C6) instead of TSA and As to prepare C6-MOF and Hm@C6-MOF. bEnd.3 cells, CTLL-2 cells and H22 cells were used as in vitro models of vascular endothelial cells, TILs and HCC cells, respectively.

**Fig. 2:**
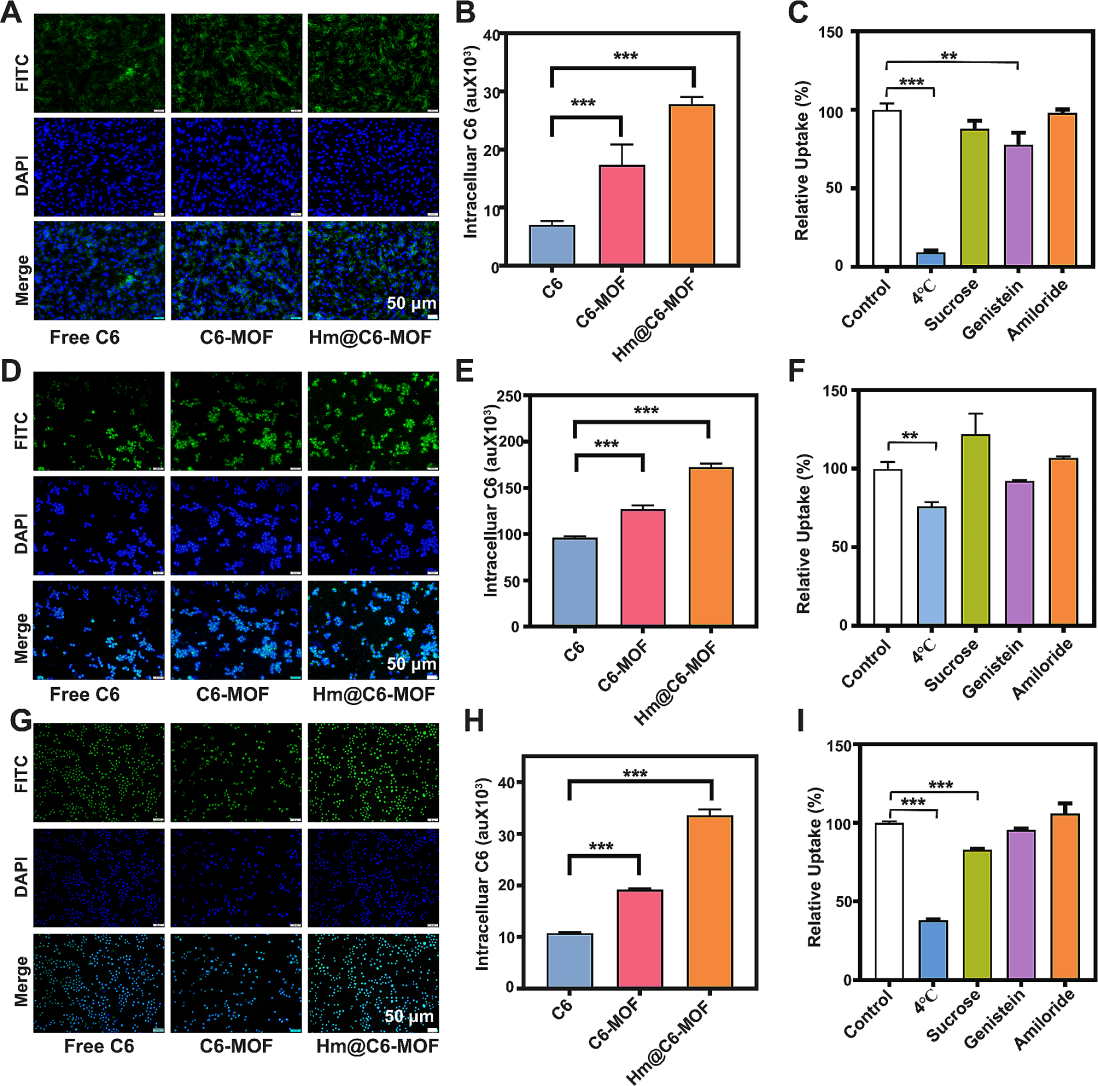
Cellular uptake of Hm@TSA/As-MOF. The uptake of free C6, C6-MOF and Hm@C6-MOF by bEnd.3 cells was examined via fluorescence microscopy (**A**) (scale bar = 50 μm) and flow cytometry (**B**). The mechanism underlying the uptake of Hm@C6-MOF by bEnd.3 cells was identified through a competitive inhibition experiment (*n* = 3) (**C**). The uptake of free C6, C6-MOF and Hm@C6-MOF by CTLL-2 cells was examined via fluorescence microscopy (**D**) (scale bar = 50 μm) and flow cytometry (**E**). The mechanism underlying the uptake of Hm@C6-MOF by CTLL-2 cells was identified through a competitive inhibition experiment (*n* = 3) (**F**). The uptake of free C6, C6-MOF and Hm@C6-MOF by H22 cells was examined via fluorescence microscopy (**G**) (scale bar = 50 μm) and flow cytometry (**H**). The mechanism underlying the uptake of Hm@C6-MOF by H22 cells was identified via a competitive inhibition experiment (*n* = 3) (**I**). Data are expressed as the mean ± SD (*, *p* < 0.05; **, *p* < 0.01; ***, *p* < 0.001; two-tailed Student’s t-test)

The results of fluorescence microscopy and flow cytometry showed that the uptake rate of Hm@C6-MOF was significantly higher than that of C6 and C6-MOF in the three cell types (Fig. 2A, B, D, E, G, H; Figure S3A, B, C). These results suggested that the uptake rate of Hm@TSA/As-MOF could be higher than TSA&As and TSA/As-MOF in vascular endothelial cells, TILs and HCC cells. This high uptake rate may be attributed to the enhanced affinity between Hm coating and cell membranes.

The mechanism underlying the cellular uptake of Hm@C6-MOF was investigated through a competitive inhibition experiment. Energy-dependent endocytosis was inhibited at 4 ℃; clathrin- and caveolae-mediated endocytosis pathways and pinocytosis was inhibited by sucrose, genistein and amiloride, respectively. The results indicated that the three types of cells endocytosed Hm@C6-MOF through an energy-dependent pathway. In particular, vascular endothelial cells endocytosed Hm@C6-MOF through caveolae-mediated endocytosis (Fig. 2C), and HCC cells endocytosed Hm@C6-MOF through clathrin-mediated endocytosis (Fig. 2I). However, TILs were not found to use any of the abovementioned pathways to endocytose Hm@C6-MOF (Fig. 2F).

Overall, the findings indicated that Hm@TSA/As-MOF could enhance the uptake of TSA and As by vascular endothelial cells, TILs and HCC cells.

### Homologous targeting and immune escape abilities of Hm@TSA/As-MOF

**Fig. 3:**
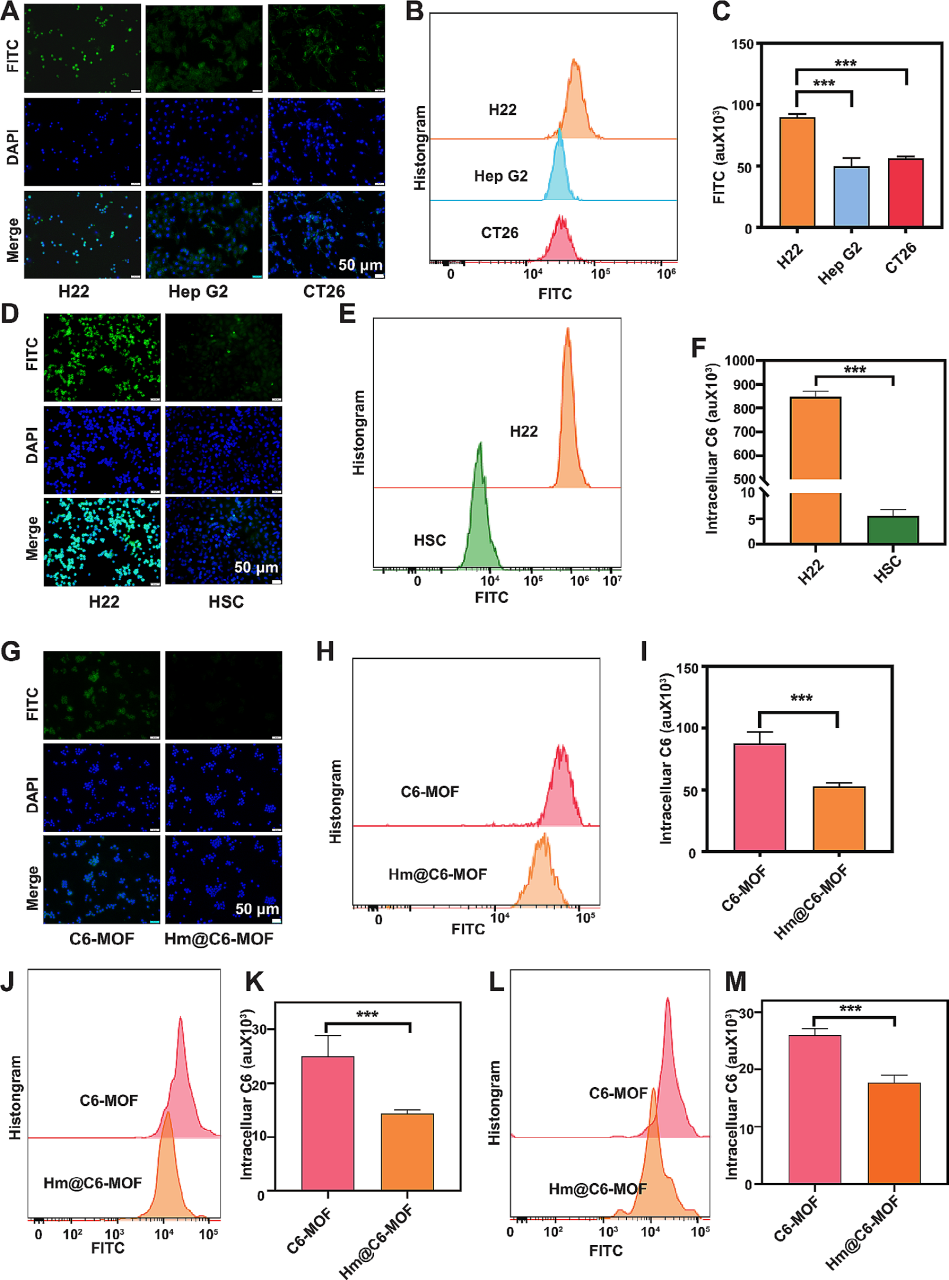
Homologous targeting and immune escape abilities of Hm@TSA/As-MOF. The homologous targeting between Hm@C6-MOF and H22, HepG2 and CT26 cells was examined via fluorescence microscopy (**A**) (scale bar = 50 μm) and flow cytometry (**B**, **C**). The homologous targeting between Hm@C6-MOF and H22 and HSC cells was examined via fluorescence microscopy (**D**) (scale bar = 50 μm) and flow cytometry (**E**, **F**). The phagocytoses of C6-MOF and Hm@C6-MOF by Raw264.7 cells were examined via fluorescence microscopy (**G**) (scale bar = 50 μm) and flow cytometry (**H**, **I**). The phagocytoses of C6-MOF and Hm@C6-MOF by macrophages in normal mice (**J**, **K**) and HCC mice (**L**, **M**) were examined via flow cytometry. Data are expressed as the mean ± SD (***, *p* < 0.001; two-tailed Student’s t-test)

Subsequently, we evaluated the homologous targeting ability of Hm@TSA/As-MOF by comparing endocytosis among CT-26, HepG2 and H22 cells. As depicted in Fig. 3A, the fluorescence signal of homotypic H22 cells was significantly stronger than that of HepG2 and CT26 cells, indicating the highly specific association between Hm@C6-MOF and H22 cells. In addition, flow cytometry revealed that endocytosis of Hm@C6-MOF was significantly more in H22 cells than in HepG2 and CT-26 cells (Fig. 3B, C). Similarly, we also found that H22 cells exhibited significantly higher uptake of Hm@TSA/As-MOF compared to normal hepatic stellate cells (HSC) (Fig. 3D, E, F). These findings suggested that Hm@TSA/As-MOF could inherit the homologous targeting ability of the H22 cell membrane, which may enhance treatment efficacy and reduce side effects in normal tissues.

Beside homologous targeting capabilities, Hm coating was also used to help nanoparticles escape the clearance by the immune system which was due to its biocompatibility. The immune evasion ability of Hm@TSA/As-MOF was evaluated using RAW264.7 cells. As anticipated, a weaker fluorescence signal was observed in RAW264.7 cells treated with Hm@C6-MOF than in those treated with C6-MOF (Fig. 3D). Flow cytometry showed that the clearance rate of Hm@C6-MOF by RAW264.7 cells in vitro was only 0.6-fold (*p* < 0.001) lower than that of C6-MOF (Fig. 3E, F). Subsequently, we investigated the immune escape behavior of Hm@C6-MOF in vivo using normal mice (Fig. 3G, H) and orthotopic mouse models of HCC (Fig. 3I, J), the results were consistent with the in vitro findings. These results suggested that the camouflage effect of Hm coating enabled Hm@TSA/As-MOF to evade clearance by MPS, thereby increasing drug bioavailability.

Altogether, coating of the H22 cell membrane can endow the Hm@TSA/As-MOF nanoplatform with both immune escape and homologous targeting abilities.

### Biodistribution of Hm@TSA/As-MOF

Based on the promising results described above, we investigated the tumour-targeting ability of Hm@TSA/As-MOF in orthotopic mouse models of HCC. To visualise and assess drug biodistribution, DiD was used to replace TSA and As and produce fluorescent signals. Subsequently, DiD, DiD-MOF and Hm@DiD-MOF were injected into mice with HCC through the tail vein. An NdFeB permanent magnet was attached to the liver area at 1 h post-administration to guide Hm@DiD-MOF to HCC tissues for 2 h.

**Fig. 4:**
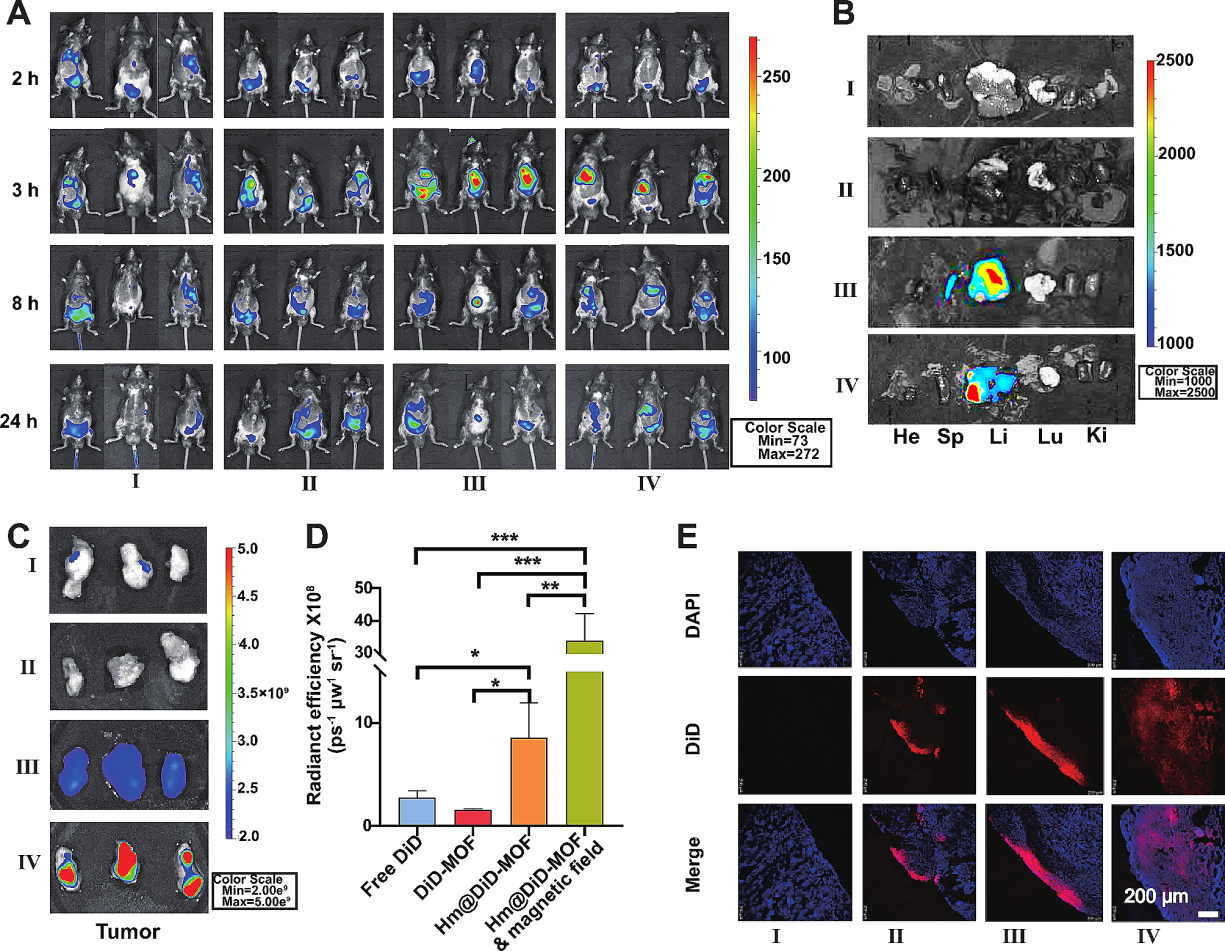
Biodistribution of Hm@TSA/As-MOF. The distribution of free DiD, DiD-MOF, Hm@DiD-MOF and Hm@DiD-MOF plus magnet in mice with HCC was visualised using an IVIS Spectrum imaging system (**A**). After 24 h, ex vivo imaging of various organs (**B**) and tumour tissues (**C**) was performed using an IVIS Spectrum imaging system. Subsequently, the ex vivo images of tumour tissues were analysed (**D**) (*n* = 3). The distribution of free DiD, DiD-MOF, Hm@DiD-MOF and Hm@DiD-MOF plus magnet in tumour tissues was examined via confocal laser microscopy (scale bar = 200 μm) (**E**) (I, DiD; II, DiD-MOF; III, Hm@DiD-MOF; IV, Hm@DiD-MOF plus magnet). Data are expressed as the mean ± SD (***, *p* < 0.001; two-tailed Student’s t-test)

In vivo NIR fluorescence images of mice at predetermined time points were shown in Fig. 4A. For mice in the Hm@DiD-MOF and Hm@DiD-MOF plus magnetic field groups, fluorescent signals were observed at liver tumour sites from 2 to 24 h after injection, with the strongest signal being recorded at 3 h after injection. Notably, the fluorescent signal of the Hm@DiD-MOF group was distributed in both the abdomen and liver tumour sites, whereas that of the Hm@DiD-MOF plus magnetic field group was mainly distributed in the liver tumour sites at 3 h after injection. This phenomenon may be attributed to the interference of ascites tumour cells with the homologous targeting of Hm coating. The guidance of a magnetic field eliminated the interference of ascites tumour cells.

Furthermore, major organs of mice were harvested for semi-quantitative analysis of biodistribution based on ex vivo imaging. In the Hm@DiD-MOF and Hm@DiD-MOF plus magnetic field groups, fluorescent signals were significantly stronger in the liver than in other organs, such as the heart and lung (Fig. 4B). Subsequently, HCC tissues were harvested from the liver for further investigation (Fig. 4C). Tumour tissues in the Hm@DiD-MOF plus magnetic field group had the highest MFI (Fig. 4 C, D), which was consistent with the results of confocal laser microscopy (Fig. 4E). This indicated that the intervention of the magnetic field could effectively avoid the interference of HCC cells in ascites with the Hm coating.

Tumour tissues in the Hm@DiD-MOF plus magnetic field group exhibited the strongest fluorescent signal, indicating that the combination of homologous targeting and magnetic guidance offers remarkable advantages in terms of tumour targeting.

### Antitumour efficacy of Hm@TSA/As-MOF combined with α-PD-1

**Fig. 5:**
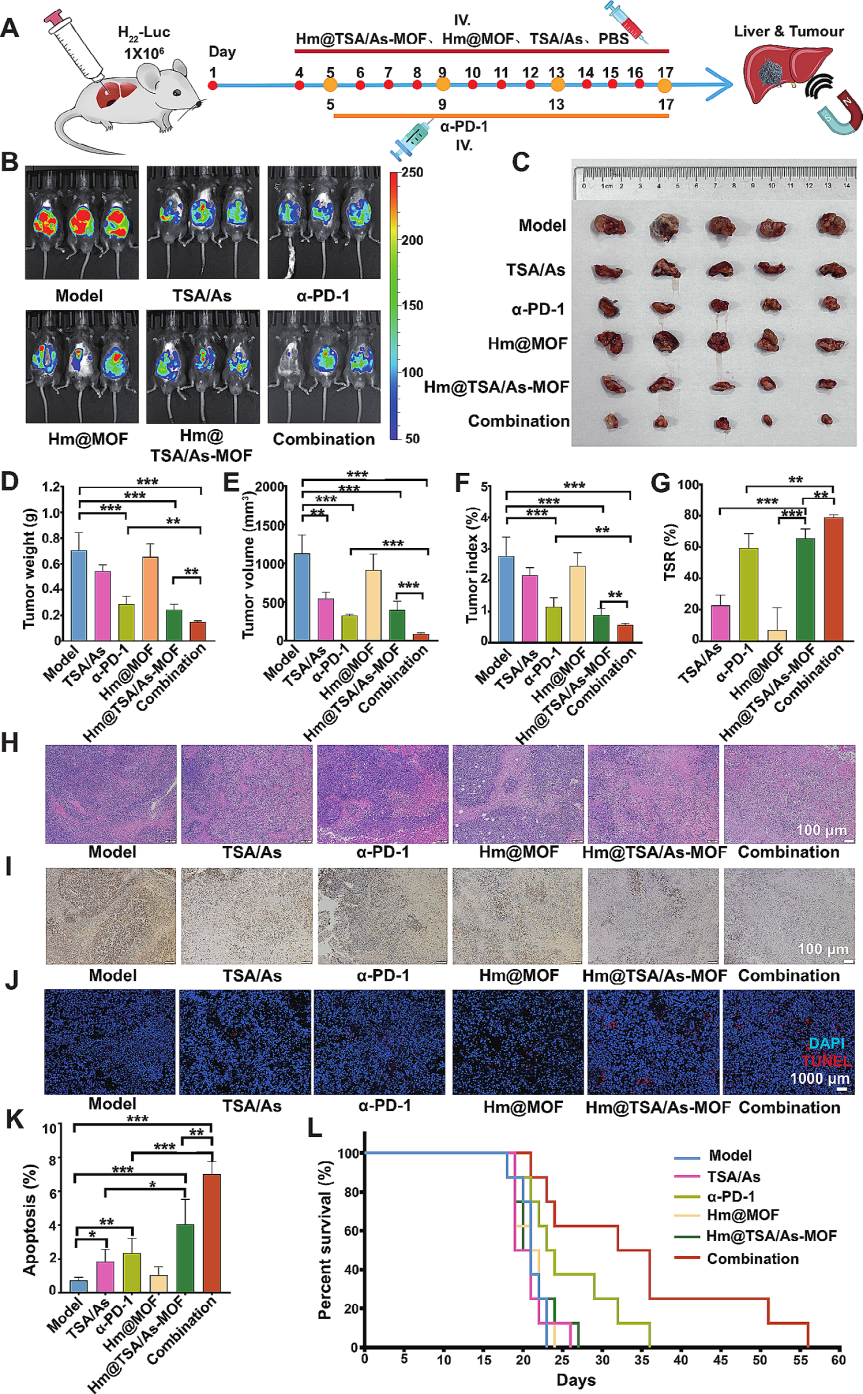
Synergistic antitumour effects of Hm@TSA/As-MOF and α-PD-1 in orthotopic mouse models of HCC. Mice with HCC were subjected to the treatment regimen illustrated in panel (**A**). Tumours were assessed via in vivo bioluminescence imaging (**B**). Representative images of tumours from each group of mice are shown (**C**). Tumour weight (**D**), tumour volume (**E**), tumour index (**F**) and TSR (**G**) were quantitatively analysed (*n* = 5). The histological structure and proliferative capacity of tumour tissues were evaluated via HE (**H**), Ki67 (**I**) and TUNEL (**J**) staining (scale bar = 100 μm). Tumour cell apoptosis was assessed via flow cytometry (**K**). The survival of mice in each group was monitored daily (**L**) (*n* = 8). Data are expressed as the mean ± SD (*, *p* < 0.05; **, *p* < 0.01; ***, *p* < 0.001 based on two-tailed Student’s t-test)

According to the abovementioned results, Hm@TSA/As-MOF can co-deliver TSA and As to the HCC microenvironment under the guidance of a magnetic field, accelerate cellular uptake, and evade clearance by MPS. To assess the therapeutic efficacy of Hm@TSA/As-MOF in combination with α-PD-1, orthotopic HCC models were established using C57BL/6 mice. Following the dosage regimen shown in Fig. 5A, mice were administered PBS, TSA and As, α-PD-1, blank carrier (Hm@MOF), Hm@TSA/As-MOF or Hm@TSA/As-MOF + α-PD-1 (combination group) *via* tail vein injection. At 1 h after injection, NdFeB was used to guide the drugs to target HCC tissues.

After 2 weeks of treatment, bioluminescence imaging was performed to examine tumours in vivo. The results revealed that the combination group exhibited the lowest bioluminescence intensity, indicating that the combination therapy effectively inhibited the growth of HCC in vivo (Fig. 5B, Figure S4). Subsequently, tumours were harvested from the livers of mice (Fig. 5C). Mice in the combination group had the lowest tumour weight, tumour volume and tumour index (Fig. 5D, E, F), suggesting that combination therapy yielded the most effective anti-HCC response. In particular, the tumour weight, tumour volume and tumour index of mice in the combination group were only 51.27% (*p* < 0.01), 26.20% (*p* < 0.001) and 49.57% (*p* < 0.01) of those in the α-PD-1 group, indicating that the combination of Hm@TSA/As-MOF enhanced the efficacy of the α-PD-1. As shown in Fig. 5G, the tumour suppression rate (TSR) of combination therapy was 79.18%, which was significantly higher than that of α-PD-1 (*p* < 0.01) or Hm@TSA/As-MOF (*p* < 0.01) monotherapy.

Furthermore, tumour tissues were sliced for histological examination. The results of H&E staining (Fig. 5H) showed the highest degree of tumour tissue necrosis in the combination group, and the results of Ki67 staining (Fig. 5I) showed the weakest proliferation of tumour tissues in the combination group. The results of TUNEL staining (Fig. 5J) and flow cytometry (Fig. 5K; Figure S5) showed that the apoptotic rates of tumour tissues were significantly higher in the combination group than in the α-PD-1 (*p* < 0.001) and Hm@TSA/As-MOF (*p* < 0.01) groups. These results suggested that combination therapy was effective in enhancing the anti-HCC effects of the α-PD-1. Additionally, mice in the combination group had the longest median survival (32 days) (Fig. 5L), indicating that combination therapy effectively prolonged the survival time of mice with HCC.

Altogether, the findings demonstrated that combination therapy with Hm@TSA/As-MOF and α-PD-1 effectively enhanced the anti-HCC effects of the α-PD-1 and improved the survival time of mice with HCC.

### Effect of Hm@TSA/As-MOF on upregulating the abundance of TILs by normalising tumour blood vessels

Given the excellent therapeutic efficacy of combination therapy with Hm@TSA/As-MOF and α-PD-1 in vivo, the mechanism underlying their synergistic anti-HCC effects was subsequently investigated.

**Fig. 6:**
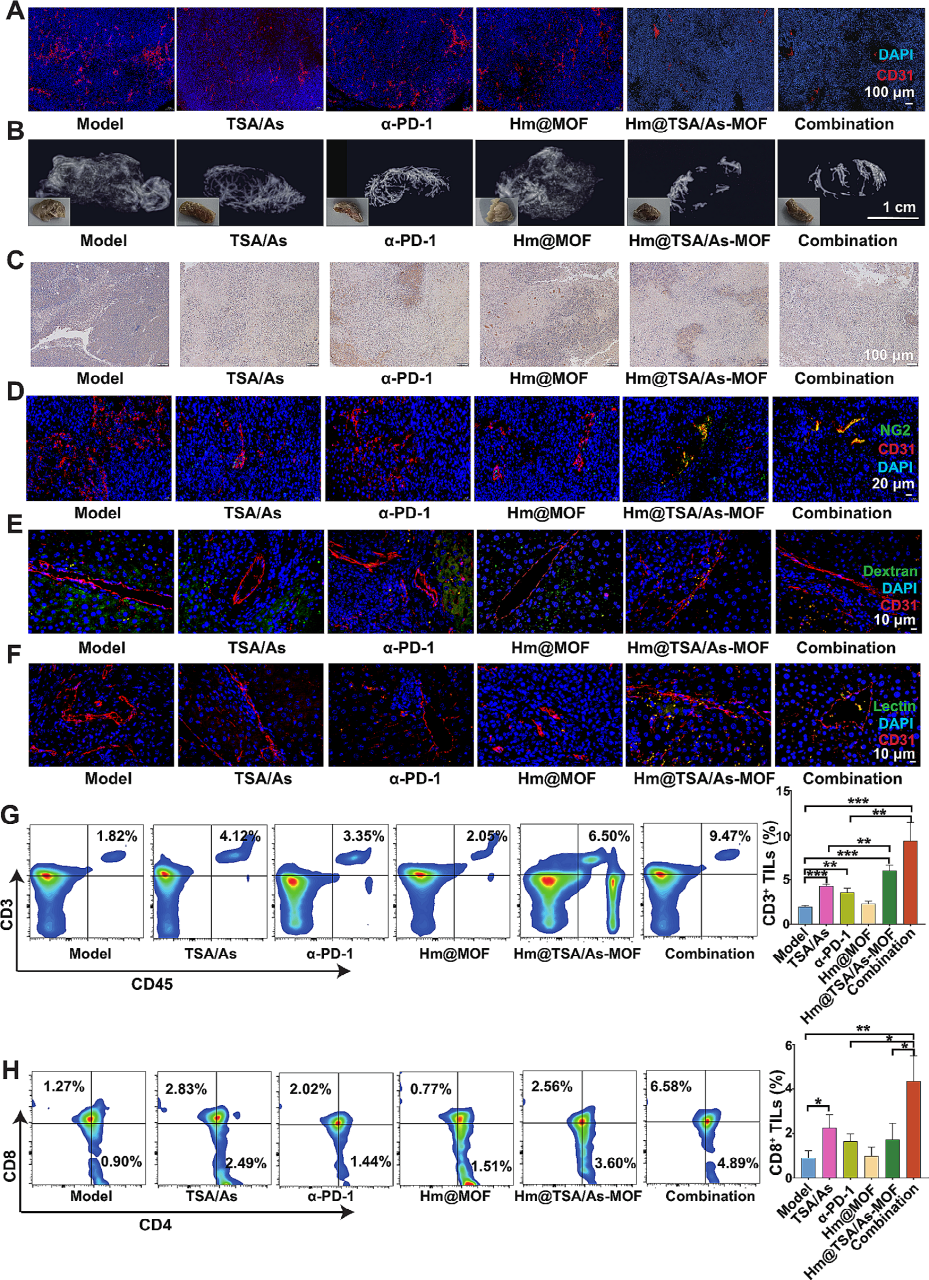
Hm@TSA/As-MOF increased the abundance of TILs by normalising tumour blood vessels. Tumour blood vessels were labelled with a CD31 antibody (scale bar = 100 μm) (**A**). The morphologies of tumour blood vessels were observed by micro CT (**B**). The levels of VEGF in tumour tissues were evaluated via immunohistochemical analysis (scale bar = 100 μm) (**C**). The pericyte coverage rate of tumour blood vessels was evaluated via NG2 staining (scale bar = 20 μm) (**D**). Dextran and lectin were used to evaluate the leakage (**E**) and perfusion (**F**) of tumour vessels, respectively (scale bar = 10 μm). The levels of CD3^+^ TILs (**G**), CD4^+^ TILs (**H**) and CD8^+^ TILs (**I**) were evaluated via flow cytometry (*n* = 3). Data are expressed as the mean ± SD (*, *p* < 0.05; **, *p* < 0.01; ***, *p* < 0.001; two-tailed Student’s t-test)

The abnormal proliferation of tumour blood vessels is considered one of the main reasons for the low abundance of TILs, which attenuates the pharmacological effects of α-PD-1 [40]. In this study, a CD31 antibody was used to label tumour blood vessels. Tumour sections from both the Hm@TSA/As-MOF and combination groups exhibited notably weaker fluorescent signals than tumour sections from the model group (Fig. 6A). Furthermore, ex vivo micro CT was used to evaluate microvessel density of tumour. The images of micro CT showed that the microvessel density of tumour in the combination group was less than α-PD-1group (Fig. 6B). Then, the results of CCK8 assay also verified that Hm@TSA/As-MOF (3.9–500 µg▪mL) effectively suppressed the proliferative activity of vascular endothelial cells (Figure S6A). Altogether, these results indicated that Hm@TSA/As-MOF and combination therapy decreased the blood vessel density of HCC tissues.

An increased level of vascular endothelial growth factor (VEGF) in the HCC microenvironment is considered the primary cause of aberrant tumour blood vessel proliferation [41, 42]. Immunohistochemical analysis (Fig. 6C) revealed that both Hm@TSA/As-MOF and combination therapy significantly reduced the levels of VEGF in tumour tissues; however, monotherapy with α-PD-1 did not demonstrate a similar capacity. These findings indicated that Hm@TSA/As-MOF decreased tumour blood vessel density by inhibiting VEGF secretion, thereby fostering an environment conducive to intra-tumoral TILs infiltration.

The characteristics of tumour blood vessels are different from those of normal blood vessels in that they have low pericyte coverage, high leakage and low perfusion owing to their disorganised structure. These characteristics can exacerbate hypoxia in the tumour microenvironment, consequently hindering the intra-tumoral TILs infiltration [12, 43–46]. In this study, an anti-NG2 antibody was used to label pericytes. The results showed that the expression of NG2 was significantly higher in the Hm@TSA/As-MOF and combination groups than in the model and α-PD-1 groups (Fig. 6D). These results suggested that tumour vessels in the Hm@TSA/As-MOF and combination groups had a higher pericyte coverage and more complete structure than those in the α-PD-1 and model groups. FITC-dextran and FITC-lectin were used to evaluate the leakage and perfusion of tumour vessels, respectively. As shown in Fig. 6E, abundant fluorescent signals for FITC-dextran were observed outside the blood vessels (labelled with CD31 antibody) in the model and α-PD-1 groups and inside the blood vessels in the Hm@TSA/As-MOF and combination groups, suggesting that Hm@TSA/As-MOF was capable of reducing vascular leakage in tumours. The results of assessment of tumour blood vessel perfusion are shown in Fig. 6F. The absence of overlap between the fluorescent signals of CD31 antibody and FITC-lectin in tumour sections from the model and α-PD-1 groups, indicating deficient tumour blood vessel perfusion, which impeded intra-tumoral TILs infiltration. However, a strong overlap between the fluorescent signals of CD31 antibody and FITC-lectin was observed in tumour sections from the Hm@TSA/As-MOF and combination groups, indicating that Hm@TSA/As-MOF enhanced tumour blood vessel perfusion in mice.

The hypoxic tumour microenvironment is an adverse consequence of low vascular perfusion, which can hinder TILs infiltration into the tumour [47]. Herein, pimonidazole (PIMO) as a probe for detecting hypoxia was intravenously injected into H22 tumour-bearing mice. The results showed that combination therapy decreased the hypoxic area in tumours, with Hm@TSA/As-MOF monotherapy exerting a similar effect (Figure S7). However, no significant reduction in the hypoxic area was observed in the α-PD-1 group, which indicated that the anti-hypoxic effects of combination therapy were mainly attributed to Hm@TSA/As-MOF.

Given that combination therapy with Hm@TSA/As-MOF and α-PD-1 promoted tumour blood vessel normalisation, we examined the effects of the combination therapy on the abundance of TILs via flow cytometry and immunofluorescence analysis. The results showed that treatment with α-PD-1 monotherapy did not lead to a significant change in the abundance of CD3^+^ TILs; however, combination therapy with Hm@TSA/As-MOF and α-PD-1 resulted in a 2.60-fold increase in the abundance of CD3^+^ TILs (*p* < 0.01) (Fig. 6G).

Among different subgroups of TILs, CD8^+^ TILs play a crucial role in eliminating tumour cells, whereas CD4^+^ TILs contribute to anti-tumour effects by assisting CD8^+^ TILs [48–50]. On quantifying CD8^+^ and CD4^+^ TIL subpopulations, we found that the abundance of CD8^+^ TILs (4.81-fold, *p* < 0.01) and CD4^+^ TILs (4.04-fold, *p* < 0.01) was significantly higher in the combination group than in the model group (Fig. 6H, Figure S8). However, no significant difference in the abundance of CD8^+^ and CD4^+^ TILs was found between the α-PD-1 and model groups. These results suggested that combination therapy increased the abundance of CD8^+^ and CD4^+^ TILs simultaneously, thereby improving the effectiveness of α-PD-1.

Altogether, the results indicated that Hm@TSA/As-MOF increased the abundance of TILs by normalising tumour blood vessels, which improved the anti-HCC effects of the combination therapy.

### Effect of Hm@TSA/As-MOF on Enhancing the Activity of TILs by Reducing the Levels of Immunosuppressive Factors

Although Hm@TSA/As-MOF was found to increase the abundance of TILs by normalising the tumour blood vessels, this effect is not sufficient for achieving the desired efficacy of combination therapy. This limited efficacy can be attributed to the presence of immunosuppressive factors in the tumour microenvironment, such as TGF-β1, IL-6 and IL-10, which suppress the activity of TILs [51–53]. 

**Fig. 7:**
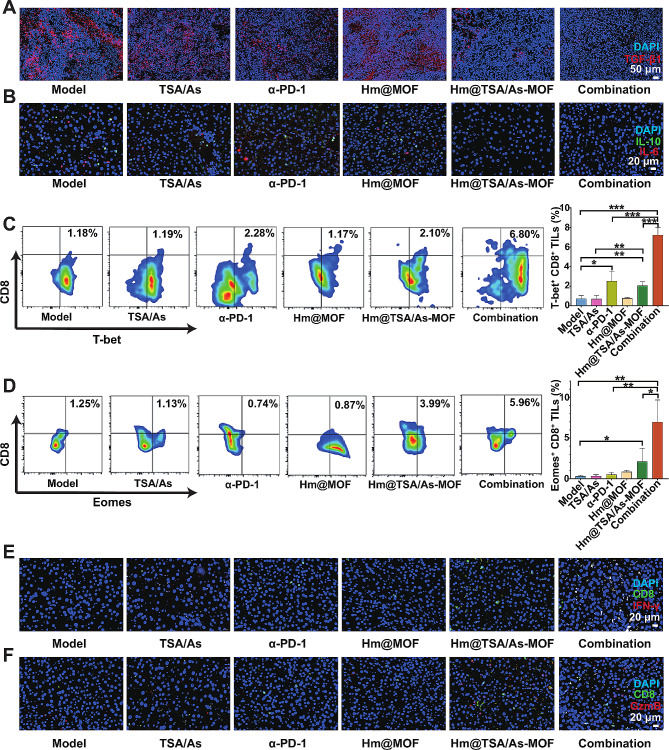
Hm@TSA/As-MOF upregulated the activity of TILs by decreasing the levels of TGF-β1. The levels of TGF-β1 (**A**), IL10 (**B**), and IL6 (**B**) in tumour tissues were evaluated via immunofluorescence staining (scale bar = 50 μm). The proportion of T-bet^+^ (**C**) and Emoes^+^ TILs (**D**) in CD8^+^ TILs was evaluated via flow cytometry (*n* = 3). The levels of IFN-γ^+^CD8^+^ TILs (**E**) and Granzyme B^+^CD8^+^ TILs (**F**) in tumour tissues were evaluated via immunofluorescence staining (scale bar = 20 μm). Data are expressed as the mean ± SD (*, *p* < 0.05; **, *p* < 0.01; ***, *p* < 0.001; two-tailed Student’s t-test)

TGF-β1 is a prototypical immunosuppressive factor known to induce TIL exhaustion and compromise the efficacy of anti-PD-1/PD-L1 therapies [51–54]. In addition, IL-6 and IL-10 also can contribute to the inhibition of the anti-tumour activity of TILs [52, 53, 55, 56]. Immunofluorescence analysis was performed to evaluate the levels of TGF-β1, IL-6 and IL-10, in HCC tissues. As shown in Fig. 7A and Fig. 7B, the combination group exhibited the least fluorescent signals of TGF-β1, followed by the Hm@TSA/As-MOF group. Notably, the fluorescent coverage of TGF-β1 was significantly lower in the combination group than in the α-PD-1 group. Similarly, the levels of IL-6 and IL-10 were lower in the combination group than in the α-PD-1 group. These findings indicated that Hm@TSA/As-MOF decreased the secretion of TGF-β1, IL-6 and IL-10, thereby mitigating the immunosuppressive microenvironment.

Subsequently, we assessed the effects of combination therapy on the activity of TILs. The results of CCK8 assay showed that Hm@TSA/As-MOF at concentrations of 15.6–31.2 µg▪mL^-1^ enhanced the proliferative activity of TILs (Figure S5B). CD8^+^ TILs are a subset of TILs mainly responsible for killing tumour cells, and T-bet and Eomes are key transcription factors involved in the anti-tumour effects of CD8^+^ TILs [57–59]. Flow cytometry showed that monotherapy with α-PD-1 increased the proportion of T-bet^+^ cells in CD8^+^ TILs, whereas monotherapy with Hm@TSA/As-MOF increased the proportion of T-bet^+^ and Emoes^+^ cells in CD8^+^ TILs. The proportion of T-bet^+^ cells and Eomes^+^ cells in CD8^+^ TILs was significantly higher in the combination group than in the α-PD-1 (T-bet^+^, *p* < 0.001; Eomes^+^, *p* < 0.01) and Hm@TSA/As-MOF (T-bet^+^, *p* < 0.001; Eomes^+^, *p* < 0.05) groups (Fig. 7 C, D). IFN-γ and granzyme B are factors secreted by CD8^+^ TILs for killing tumour cells [60]. Immunofluorescence analysis showed that monotherapy with α-PD-1 increased the levels of IFN-γ and granzyme B to a certain degree, which is consistent with the findings of a study by Shi et al. [61] However, the regulatory effects of α-PD-1 on IFN-γ and granzyme B were limited, attenuating its ability to achieve the desired therapeutic efficacy. The levels of IFN-γ and granzyme B were higher in the combination group than those in the α-PD-1 group (Fig. 7E, F). These findings suggested that combination therapy with Hm@TSA/As-MOF and α-PD-1 enhanced the anti-tumour activity of CD8^+^ TILs.

Altogether, the abovementioned results showed that Hm@TSA/As-MOF enhanced the anti-tumour activity of TILs and improved the anti-HCC effects of α-PD-1 by decreasing the levels of TGF-β, IL-6 and IL-10 in the HCC microenvironment.

### Safety of combination therapy with Hm@TSA/As-MOF and α-PD-1

Safety is a noteworthy advantage of immunotherapy when compared with chemotherapy and radiation therapy [62, 63]. Therefore, it is necessary to ensure that combination therapy with Hm@TSA/As-MOF and α-PD-1 does not introduce any supplementary safety concerns.

**Fig. 8:**
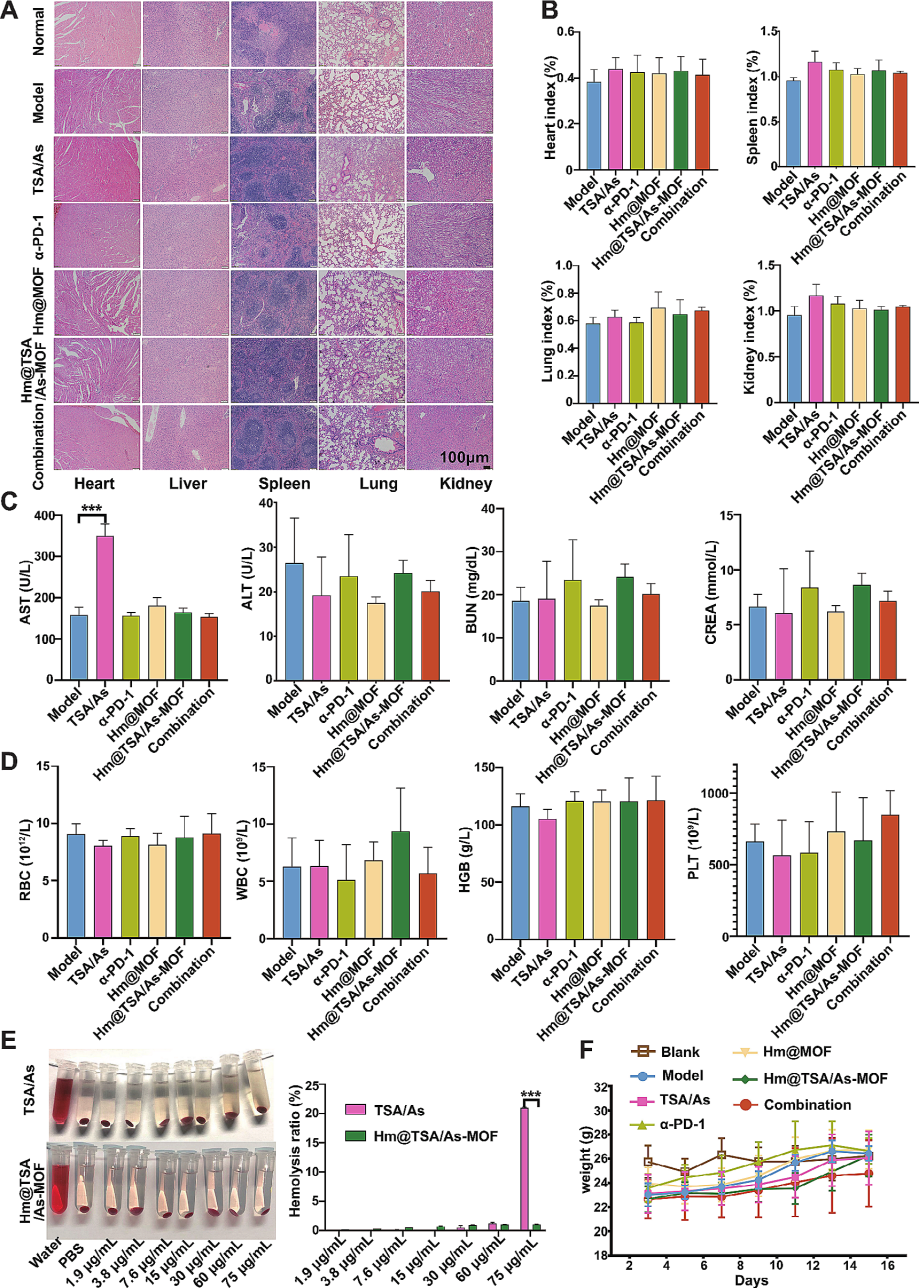
Evaluation of the safety of Hm@TSA/As-MOF. Tissue slices of internal organs were used to observe the effects of drugs in mice (**A**). The heart, spleen, lung and kidney indices of mice were evaluated and statistically analysed (*n* = 5) (**B**). AST, ALT, BUN and CREA levels were evaluated to assess the liver and kidney function of mice (*n* = 3) (**C**). The RBC count, WBC count, HGB level and PLT count of mice were evaluated and statistically analysed (*n* = 5) (**D**). The haemolytic properties of TSA&As and Hm@TSA/As-MOF were tested and statistically analysed (*n* = 3) (**E**). The body weight of mice in each group was monitored and recorded during treatment (*n* = 8) (**F**). Data are expressed as the mean ± SD (***, *p* < 0.001; two-tailed Student’s t-test)

At the end of the treatment, we assessed the safety of the combination therapy using tissue sections and organ indices. The results showed no observable tissue damage in the organs of the mice within the combination group (Fig. 8A), and no significant differences in organ indices were observed among the combination, Hm@TSA/As-MOF, α-PD-1 and model groups (Fig. 8B). Furthermore, biochemical analysis revealed that the combination therapy neither induced hepatotoxicity or nephrotoxicity (Fig. 8C) nor led to any alterations in blood routine parameters (Fig. 8D).

As a saponin, astragaloside IV is associated with a risk of haemolysis theoretically. Therefore, we evaluated the haemolytic effects of TSA&As and Hm@TSA/As-MOF. The results revealed that TSA&As led to pronounced haemolysis, whereas Hm@TSA/As-MOF did not exert a similar effect (Fig. 8E). This result suggested that Hm@TSA/As-MOF has the potential to reduce the hemolytic risk associated with astragaloside IV.

To assess the impact of the combination therapy on the quality of life, we monitored the body weight of mice throughout the treatment period. The results indicated no discernible differences in body weight among any of the groups (Fig. 8F), suggesting that the combination therapy did not impose any additional burden on the body of mice.

Altogether, the results revealed that the combination therapy had a credible safety profile.

## Conclusion

In this study, we developed a biomimetic–magnetic dual-targeting nanoplatform composed of magnetic MOFs and homologous tumour cell membrane coating (Hm@TSA/As-MOF), which was used to co-deliver TSA and As into the HCC microenvironment to enhance the anti-HCC effects of α-PD-1 therapy. Hm@TSA/As-MOF exhibited a spherical shape with a size of 248.60 ± 16.20 nm and had a high total DLC (15.3 wt%). It was readily internalised by vascular endothelial cells, TILs and HCC cells, and could evade the immune clearance by MPS. In addition, it efficiently co-delivered TSA and As to HCC tissues through biomimetic–magnetic dual-targeting, and exhibited controlled release of TSA and As in response to the reductive and acidic microenvironment of HCC. Mechanistically, Hm@TSA/As-MOF simultaneously increased the abundance and activity of TILs by normalising the tumour blood vessels and reducing the levels of immunosuppressive factors, respectively. When used in combination with an α-PD-1, Hm@TSA/As-MOF improved the overall anti-HCC effect, and had a credible safety profile. In conclusion, this study introduces a novel strategy for co-delivering multiple drugs to the tumour site to improve the therapeutic efficacy of PD-1 inhibitors in clinical settings.

## Reagents and methods

### Reagent

Tanshinone II_A_, astragaloside IV, and sodium acrylate were purchased from Yuanye Biotechnology Co. Ltd. (Shanghai, China). FeCl_3_[]6H2O, diethylene glycol, and DiD were purchased from Aladdin Reagent Co., Ltd. (Shanghai, China). Benzene-1, 3, 5-tricarboxylic acid (BTC) was purchased from Yien Chemical Technology Co. Ltd. (Shanghai, China). HNO_3_, HF, and sodium acetate anhydrous were purchased from Nanjing Chemical Reagent Co. Ltd. (Nanjing, China). CCK-8 kit was purchased from Dojindo Laboratories Co. Ltd. (Kyushu, Japan). RIPA lysis buffer and BCA kit were purchased from Jiangsu Keygen Biotech Co. Ltd. (Jiangsu, China). InVivoMAb Anti-mouse PD-1 (CD279) (Lot: 84,292,201; Coln: RMP1-14; Cat: BE0146) were purchased from BioXCell (Florida, America). Anti-mouse VEGF (GB15165-100), anti-mouse CD31 (GB12063-100) and anti-mouse NG2 (GB115534-100) were purchased from Servicebio Technology Co. Ltd. (Wuhan, China). Brilliant Violet 421™ anti-mouse CD45 (103,133), FITC anti-mouse CD4 antibody (100,405), and Alexa Fluor® 700 anti-mouse CD8a (100,730) were purchased from Biolegend Co., Ltd. (California, America). IFN gamma monoclonal antibody, PE/Cyanine7 anti-mouse CD3 (25-0032-82), PE/eomes menocional antibody (35-4877-42), and APC/T-bet monoclonal antibody (17-5825-80) were purchased from eBioscience Co., Ltd. (California, America). BaSO_4_ was purchased from Macklin Co., Ltd. (Shanghai, China). All chemicals were used directly without further purification.

### Cell lines

bEnd.3 (TCM40) was obtained from the National Collection of Authenticated Cell Culture (Shanghai, China). CTLL-2 (STCC20042P), and H22 (STCC20036P) cell were obtained from the Servicebio Technology Co. Ltd. (Wuhan, China). H22-Luc cell was provided by the China Pharmaceutical University (Nanjing, China). bEnd.3, CTLL-2, H22, and H22-Luc cell were cultured in a cell incubator at 37 °C under 5% CO_2_. The medium of bEnd.3 was DMEM with 10% fetal bovine serum (FBS), and the mediums of CTLL-2, H22, and H22-Luc were RPMI 1640 with 10% FBS.

### Animals

Male C57BL/6 mice (6–8 weeks old, 18–20 g) were purchased by GemPharmatech LLC. (Nanjing, China; SCXK(SU)2023-0009). All animal procedures were performed in accordance with the Guidelines for Care and Use of Laboratory Animals of Jiangsu Province Academy of Traditional Chinese Medicine (Nanjing, China; SYXK(SU)2021-0025).

### Preparation of Fe_3_O_4_ nanoparticles

In a typical process, 1.5 g sodium acetate anhydrous, 1.5 g sodium acrylate, 0.75 g FeCl_3_▪6H_2_O and 40 mL diethylene glycol were mixed together and stirred at 400 r▪min^-1^ for 12 h. Then, the solution was enclosed in a teflon-lined autoclave for heating at 190 ℃ for 10 h to obtain the Fe_3_O_4_ nanoparticles. The nanoparticles were washed by ethanol for 3 times, and then they were centrifuged at 55,000 r▪min^-1^ for 30 min. The precipitates were retained as Fe_3_O_4_ nanoparticles.

### Preparation of MOF

100 mg Fe_3_O_4_ nanoparticles and 287.5 mg BTC were dispersed into 50 mL of acid solution with 0.01 mM▪mL^-1^ HNO_3_ and 0.003 mM▪mL^-1^ HF. Next, the solution was transferred into a teflon-lined autoclave for heating at 150 °C for 4 h to obtain the MOF. The MOF was washed with ethanol/water at 60 ℃ for 2 times. Finally, the MOF was collected by a magnet.

### Drug loading

40 mg TSA and 25.8 mg As were dissolved into 40 mL methanol. Then, 10 mg MOF was added into the solution of TSA and As. The solution was stirred at 150 r▪min^-1^ for 12 h in room temperature. After stirring, TSA/As-MOF was collected by a magnet.

### Preparation of Hm

H22 cells were suspended in PBS containing protease inhibitor, and broken repeatedly using a freeze-thaw method. The solution was centrifugated at 700 g for 10 min in 4 ℃, then, the supernatant was subjected to further centrifugation at 14,000 g for 30 min to collect the cell membrane (Hm).

### Preparation of Hm@TSA/As-MOF

Hm was mixed with TSA/As-MOF according to 1:2 (w: w) in water, then, the mixture was sonicated at 200 w for 10 min in 4 ℃. Finally, Hm@TSA/As-MOF was collected by a magnet.

### Measurements and characterizations

The size and zeta potential of the nanoplatform was determined by a zeta potential/particle sizer (Zetasizer Nano ZS, Malvern Panalytical). The morphology of the nanoplatform was examined by a SEM (LEO 1530VP, LEO Election Microscopy Ltd.) and a TEM (Tecnai G2 F20 S-Twin, FEI). Magnetic properties were determined by using a VSM (LakeShore7404, Lake Shore) and a NdFeB magnet. Crystal characteristics were determined by using an XRD (Max-2200PC, Rigakud). The IR spectra of the nanoplatform was determined by a FTIR (Spectrum GX, Perkin-Elmer). The UV spectra of the nanoplatform was determined by a UV (UV1800PC, Phenix). The thermogravimetric analysis was tested in a N_2_ environment by a TG-DSC (PE, Mettler Toledo).

### DLE

DLE was determined by HPLC. The contents of Om and As in the methanol solution before (M1) and after (M2) drug loading were determined by HPLC. DLE was calculated by the ratio between the (M1 - M2) and the mass of Hm@TSA/As-MOF.

TSA was detected on an Agilent 1260 HPLC with an ODS2 column (5 μm, 4.6 mm × 150 mm, Elite, Dalian, China). The column temperature set at 30 ℃, the injection volume was 5 µL, and the flow rate of the mobile phase was 1 mL▪min^-1^. The elution gradient of mobile phase contained 82% solvent A (acetonitrile) – 18% solvent B (water), and the elution time was 10 min. Using a UV detector for detection, with a detection wavelength of 270 nm.

As was detected on an Agilent 1260 HPLC with an ODS2 column (5 μm, 4.6 mm × 150 mm, Elite, Dalian, China). The column temperature set at 35 ℃, the injection volume was 10 µL, and the flow rate of the mobile phase was 1 mL▪min^-1^. The elution gradient of mobile phase contained 35% solvent A (acetonitrile) – 65% solvent B (water), and the elution time was 10 min. Using an ELSD detector for detection, with a drift tube temperature of 50 °C; carrier gas flow rate of 1.8 L▪min^-1^; gain coefficient of 1.

### Drug release

Hm@TSA/As-MOF was placed in 4 solutions (neutral solution: pH7.4, 0.5% SDS-PBS; acidic solution: pH6.4, H_3_PO_4_-0.5% SDS-PBS, reductive solution: 10 mM GSH-0.5% SDS-PBS, reductive and acidic solution: 10 mM GSH-pH6.4, H_3_PO4-0.5% SDS-PBS) for drug release studies. Solutions were collected at different time periods and replenished with fresh solutions. The collected solutions were concentrated 5× and detected by HPLC.

### Hm protein characterization

Protein characterization was conducted using sodium dodecyl sulfate-polyacrylamide gel electrophoresis (SDS-PAGE) method. Firstly, the Hm and Hm@TSA/As-MOF were lysed in RIPA lysis buffer containing protease inhibitor cocktail and phosphatase inhibitor cocktail on ice for 5 min. Then, the lysates were centrifuged at 13,000 g for 5 min at 4 ºC, the supernatant was then subjected to enhanced BCA protein assay for the quantification of the total protein. After that, the protein was mixed with loading buffer and heated at 100 ºC for 5 min. An equivalent of 30 µg of total protein per sample was loaded into each well of an 8% tris/glycine SDS-poly-acrylamide gelatin in an electrophoresis chamber system. Finally, the protein blot was stained with a protein staining kit (EnoGene).

### Fluorescently labeled Hm@TSA/As-MOF

5 mg C6 was dissolved in 10 mL methanol and sonicated 10 min at room temperature. Follow the method in “*Preparation of Hm@TSA/As-MOF*” to get C6-MOF and Hm@C6-MOF; 5 mg DiD was dissolved in 5 mL methanol and sonicated 10 min at room temperature. Follow the method in “*Preparation of Hm@TSA/As-MOF*” to get DiD-MOF and Hm@DiD-MOF. All the above operations were performed in a dark environment.

### Cellular uptake and immune escape

bEnd.3, CTLL-2, H22, and Raw264.7 cell were seeded in a 6-well cell culture plate at a density of 1 × 10^5^ cells/well and then incubated at 37 °C overnight. After different treatments, 4 °C PBS was used to terminate cellular uptake, and DAPI was used to label cells. Fluorescence microscopy (IX-73, Olympus) was used to observe the cellular uptake of different drugs. At the same time, cells were collected and counted the brightness of the fluorescence using flow cytometry (CytoFLEX, Beckman).

In vivo immune escape was testes in normal mice and HCC mice. C6-MOF and Hm@C6-MOF were injected intravenously into normal mice and HCC mice. After 4 h, the spleens of the mice were collected and processed into a single cell state. Anti-mouse F4/80 and anti-mouse CD11b were used to label macrophages, and flow cytometry was used to measure the efficiency of macrophages to phagocytose nanoparticles.

The mechanism of cellular uptake was reflected by competitive inhibition experiments. Briefly, after the cells were treated with sucrose, genistein, amiloride, and a 4 °C environment, Hm@C6-MOF was used to co-incubate with the cells. Further, flow cytometry was used to determine the efficiency of drug uptake by cells.

### Cellular homologous targeting

H22, Hep G2, and CT26 cells were seeded in a 6-well cell culture plate at a density of 1 × 10^5^ cells/well and then incubated at 37 °C overnight. After different treatments, 4 °C PBS was used to terminate cellular uptake, and DAPI was used to label cells. Fluorescence microscopy was used to observe the cellular uptake. At the same time, cells were collected and counted the brightness of the fluorescence using flow cytometry.

### Orthotopic transplantation tumour model

Mice were randomly divided into different subgroups. 1 × 10^6^ H22-Luc cells were transplanted into the liver of C57BL/6 mouse. Bioluminescence imaging was performed using an IVIS Spectrum (PerkinElmer) to exclude the mice that have no tumour. The mice that have tumour were randomly assigned to different groups.

### In vivo fluorescence imaging study

Equal doses of DiD, DiD-MOF, and Hm@DiD-MOF were injected into the mice with HCC *via* the tail vein. Images were captured at different postinjection times (2, 3, 8, and 24 h) by IVIS Spectrum. At 24 h after injection, mice were sacrificed, and organs (heart, spleen, lung, and kidney) and tumours were harvested for ex vivo fluorescence imaging. Laser confocal microscopy (TCS SP8, Leica) was used to observe the distribution of drugs in HCC tissue.

### In vivo antitumour efficacy

Orthotopic transplantation model of HCC in C57BL/6 mice (*n* = 8/each group) was established according to the description in “*Orthotopic Transplantation Tumour Model*”. After 3 days, mice were treated respectively with PBS, TSA&As (TSA: 0.8 mg▪Kg^-1^; As: 0.2 mg▪Kg^-1^), blank carrier (15 mg▪Kg^-1^), and Hm@TSA/As-MOF (15 mg▪Kg^-1^) *via* intravenous administration every day. Mice in the α-PD-1 group and the combination group were injected with α-PD-1 (5 mg▪Kg^-1^) *via* intravenous administration every 4 days. At the end of the antitumour efficacy study, mice were sacrificed, and tumours were collected as well as weighed, photographed, and sectioned. Slices were stained through HE, Ki67, and TUNEL to observe the histopathological changes. Apoptosis of tumour was measured using flow cytometry. Survival experiment was also performed. Besides, ascites was collected and studied. Tumor suppression rate (%) was calculated as following Eq.

Tumor suppression rate (%) = [(Ac - Ax) / Ac] × 100% [64].

Where A c and A x represented the average weight of tumors in the control (Model) and the treatment (TSA/As, α-PD-1, Hm@MOF, Hm@TSA/As-MOF and combination) groups.

### Flow cytometry analysis

Tumour tissues were digested at 37 °C in a buffer containing 1 mg▪mL^-1^ collagenase I, 1 mg▪mL^-1^ collagenase IV, 0.04 mg▪mL^-1^ DNAase and 0.4 mg▪mL^-1^ HAase for 5 h. Subsequently, the mixtures were filtered to collect single cells. Single cells were labeled with antibodies and analyzed using flow cytometry.

### Electronic supplementary material

Below is the link to the electronic supplementary material.

Supplementary Material 1

## Data Availability

All data presented in this paper are included in the main text and the Additional file.
