# Supplementary material for: Homologous-magnetic dual-targeted metal-organic framework to improve the Anti-hepatocellular carcinoma efficacy of PD-1 inhibitor
Source: J Nanobiotechnology. 2024 Apr 24;22:206. doi: 10.1186/s12951-024-02469-6 (PMC11044376; doi:10.1186/s12951-024-02469-6)
Supplement: Supplementary file 1 — Supplementary Material 1 [file 12951_2024_2469_MOESM1_ESM.docx]

Supporting Information

Biomimetic-Magnetic Dual-Targeted Metal-Organic Framework to Improve the Anti-Hepatocellular Carcinoma Efficacy of PD-1 Inhibitor

Hong Guo ^‡^, Xia Li ^‡^, Dengxuan Mao, Hong Wang, Liangyin Wei, Ding Qu, Xiaoying Qin, Xiaoqi Li, Yuping Liu*, and Yan Chen*.


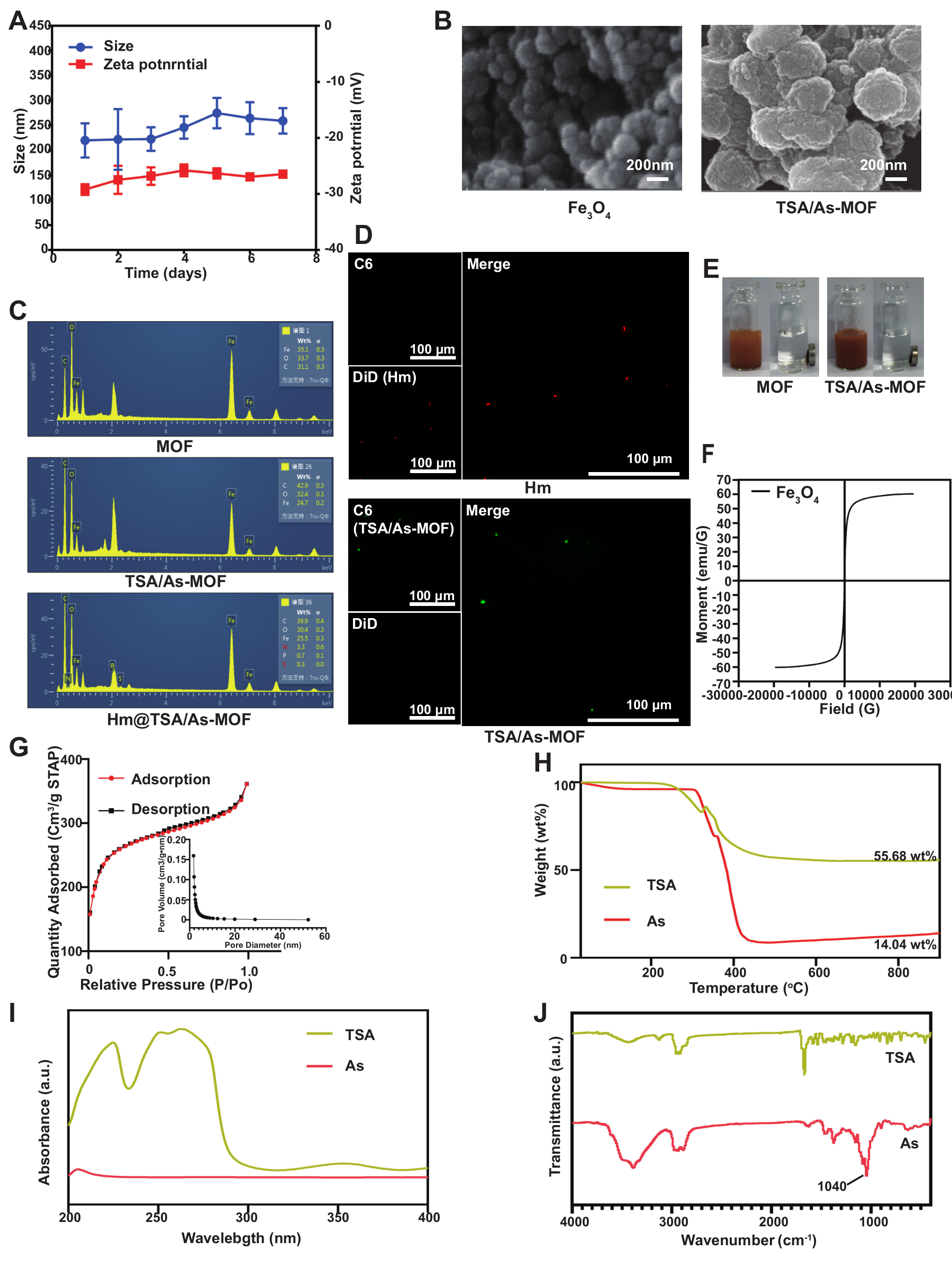


**Figure S1**. Changes in Hm@TSA/As-MOF size and zeta potential were monitored over the course of a week using DLS (A). Image demonstrating morphologies of Fe_3_O_4_ nanoparticles and TSA/As-MOF taken by SEM (scale bar = 200 nm) (B). Elemental composition analysis of MOF, TSA/As-MOF, and Hm@TSA/As-MOF were conducted using SEM coupled with energy-dispersive X-ray spectroscopy (SEM-EDS) (C). The Hm and TSA/As-MOF were demonstrated by laser confocal microscopy, respectively (D). The responsiveness of MOF and TSA/As-MOF to magnetic fields were evaluated using NdFeB permanent magnets, respectively (E). The responsiveness of Fe_3_O_4_ to magnetic fields was tested using VSM (F). The pore characteristics of MOF was observed by the curves of nitrogen adsorption and desorption (G). The TGA (H), UV (I), and FTIR (J) results of TSA and As are shown.


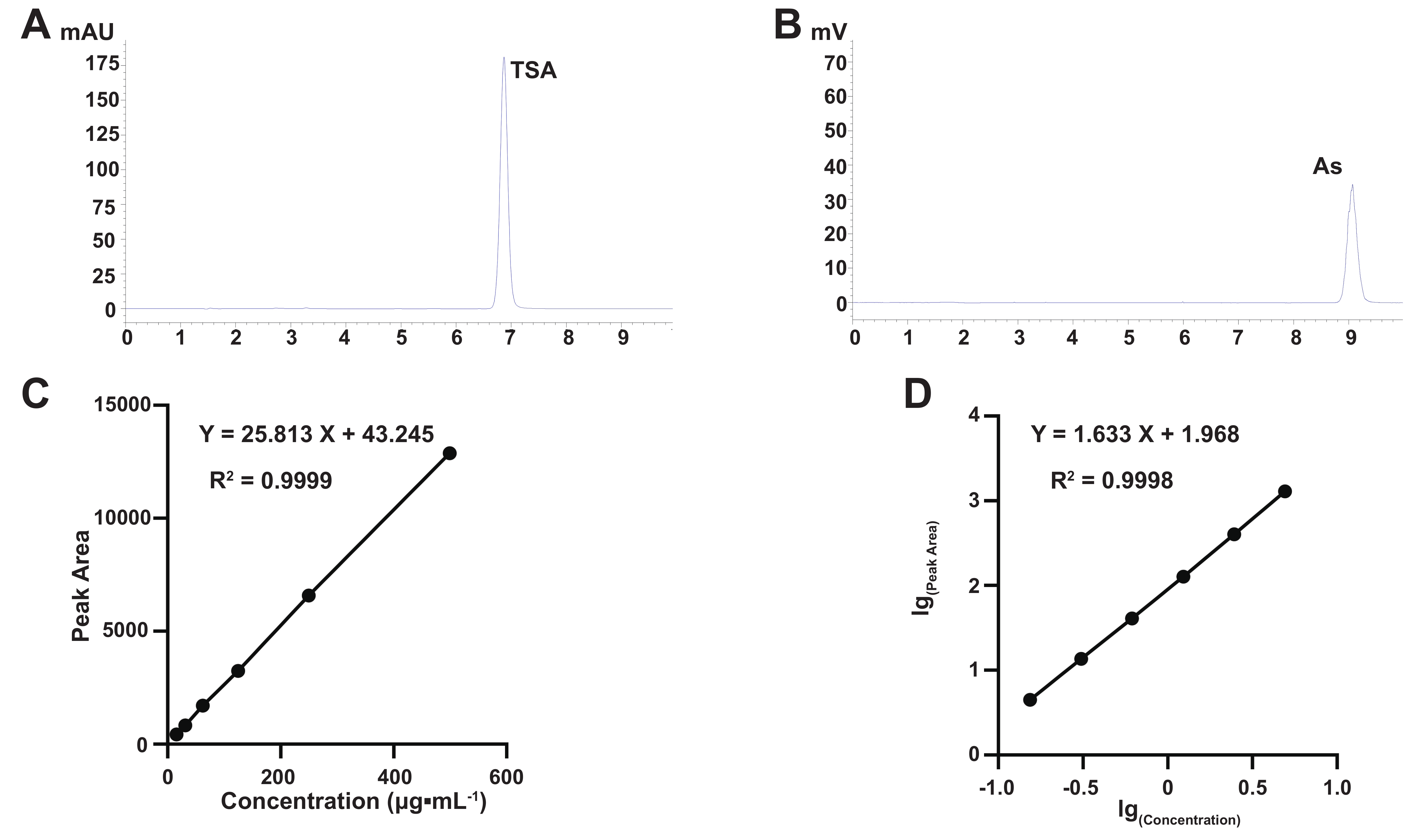


**Figure S2**. The HPLC chromatograms of TAS (A) and As (B), and the standard curves of TAS (C) and As (D).


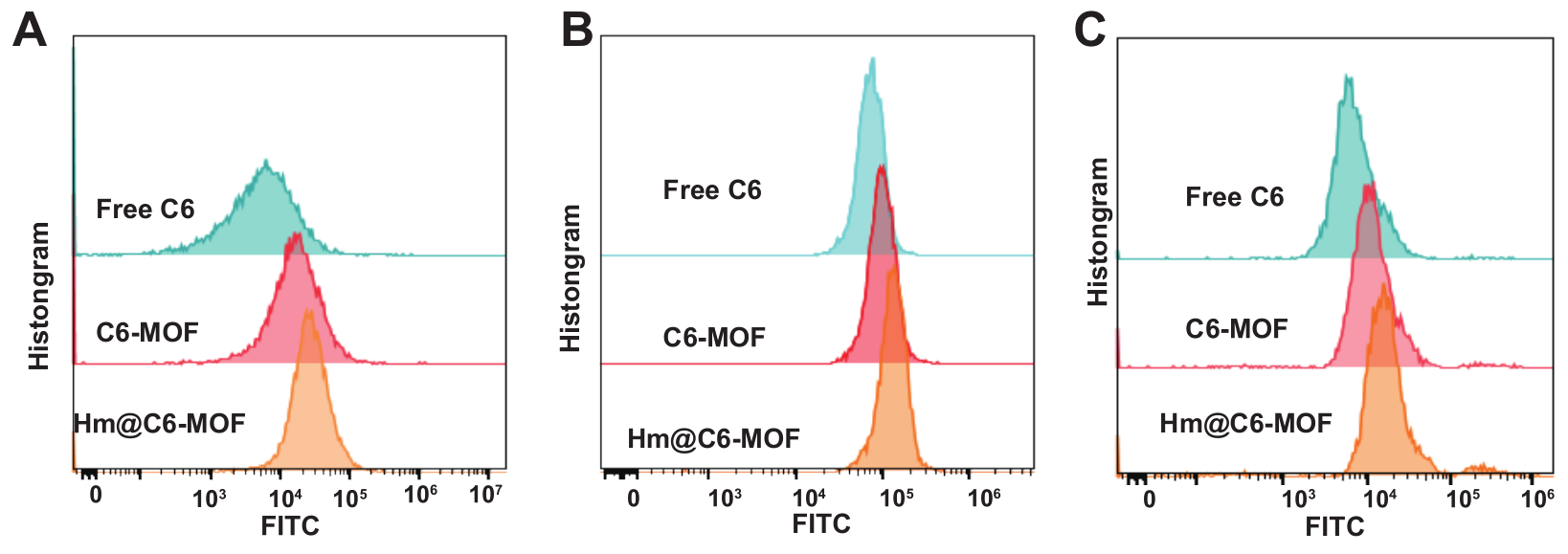


**Figure S3**. Uptake analysis of free Free C6, C6-MOF, and Hm@C6-MOF by bEnd.3 cells (A), TILs (B), and HCC cells (C) *in vitro* (n=3). Data represent the means ± SD. (**, *p* < 0.01; ***, *p* < 0.001; two-tailed Student’s t-test).


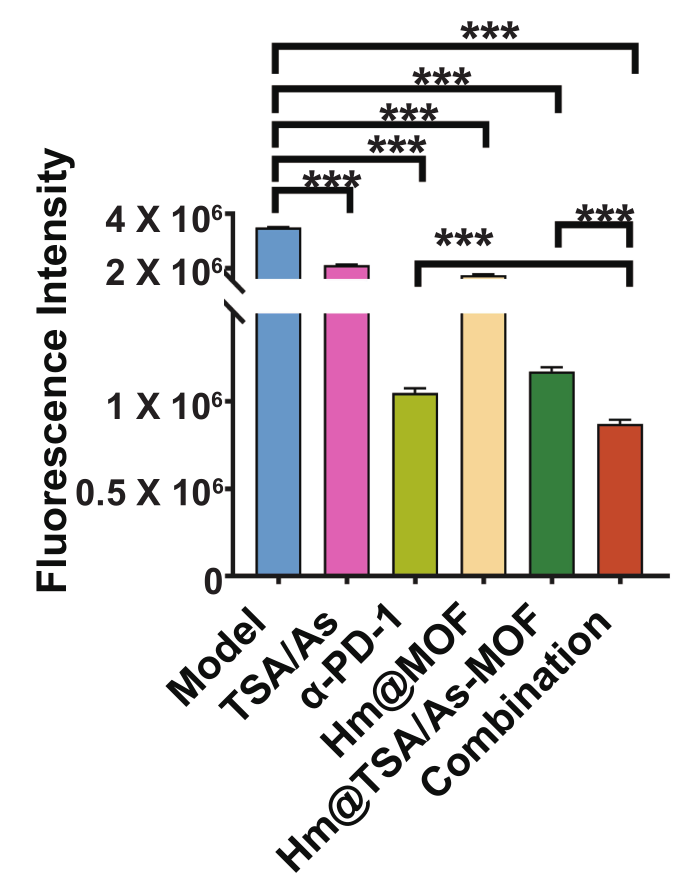


**Figure S4**. The extent of tumor bioluminescence in mice was measured and analyzed (n=6). Data represent the means ± SD. (***, *p* < 0.001; two-tailed Student’s t-test).


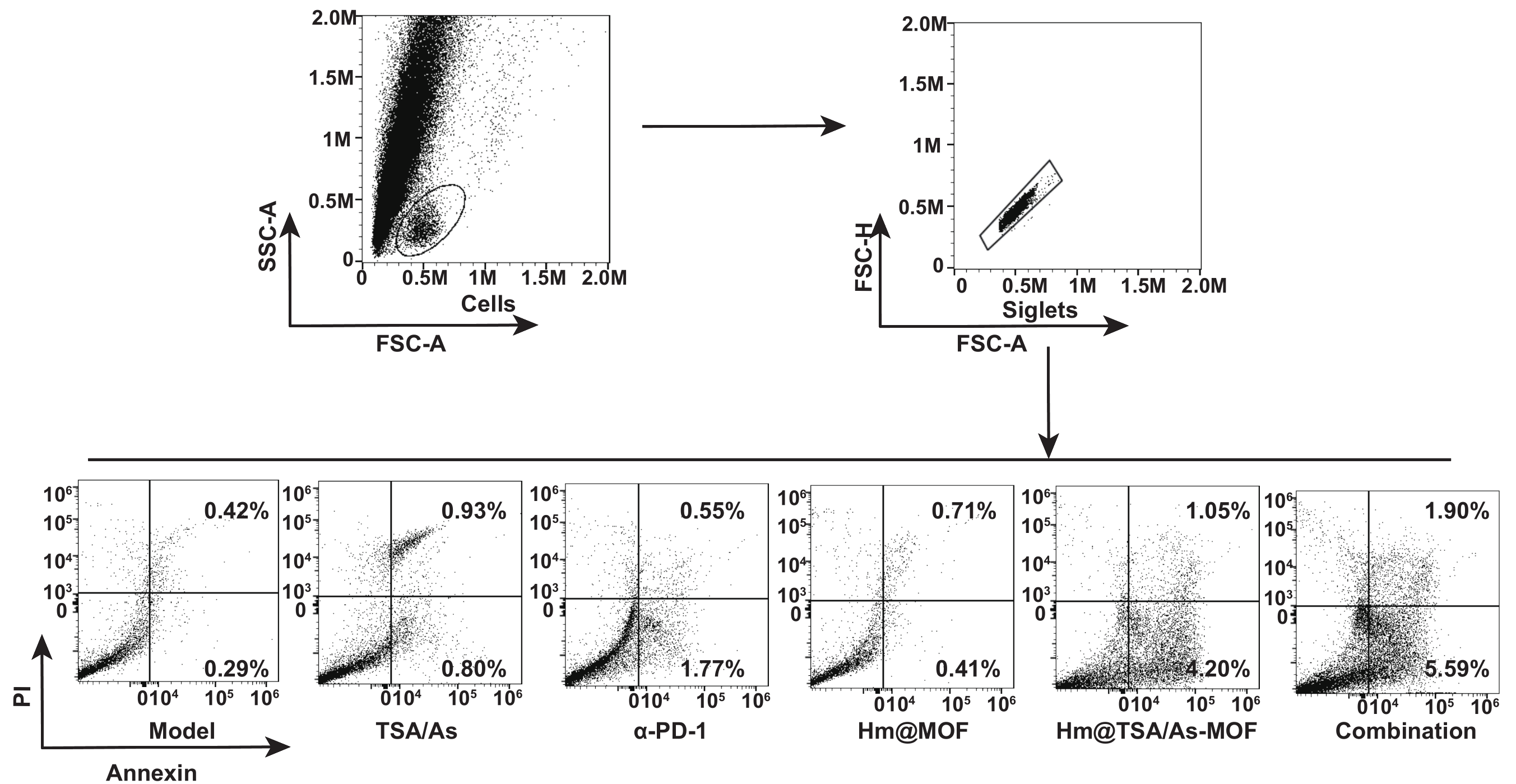


**Figure S5**. Tumor cell apoptosis was assessed by flow cytometry.


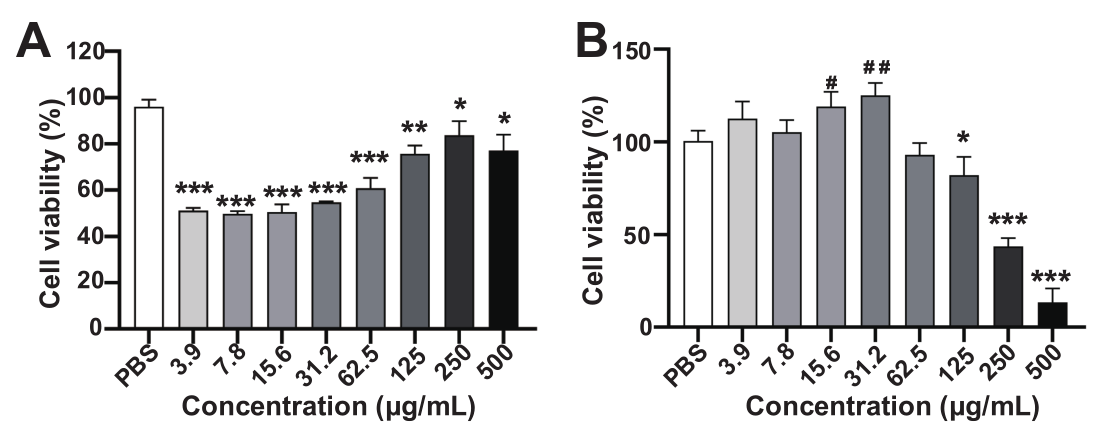


**Figure S6**. *In vitro* assessment of the effects of Hm@TSA/As-MOF on the activity of bEnd.3 (A) and TILs (B) using CCK-8 assay (n=6). Data were presented as means ± SD. (inhibition of viability: *, *p* < 0.05; **, *p* < 0.01; ***, *p* < 0.001; enhancement of viability: ^#^*p* < 0.05, ^##^*p* < 0.01; vs. PBS two-tailed Student’s t-test).


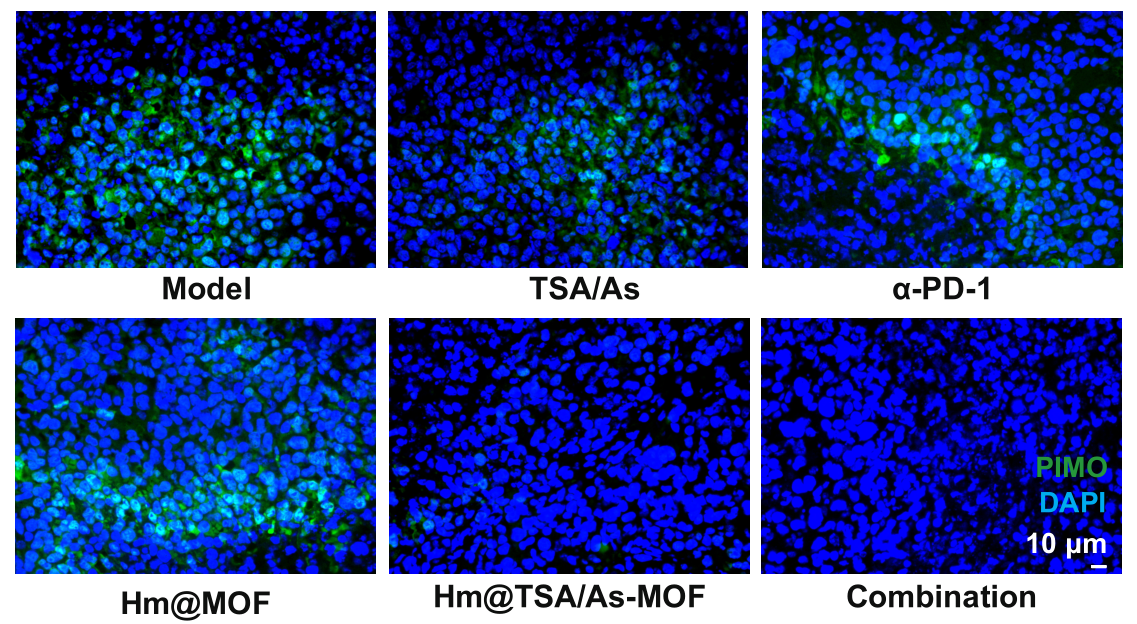


**Figure S7**. Tumour hypoxia was assessed using PIMO (scale bar = 10 μm).


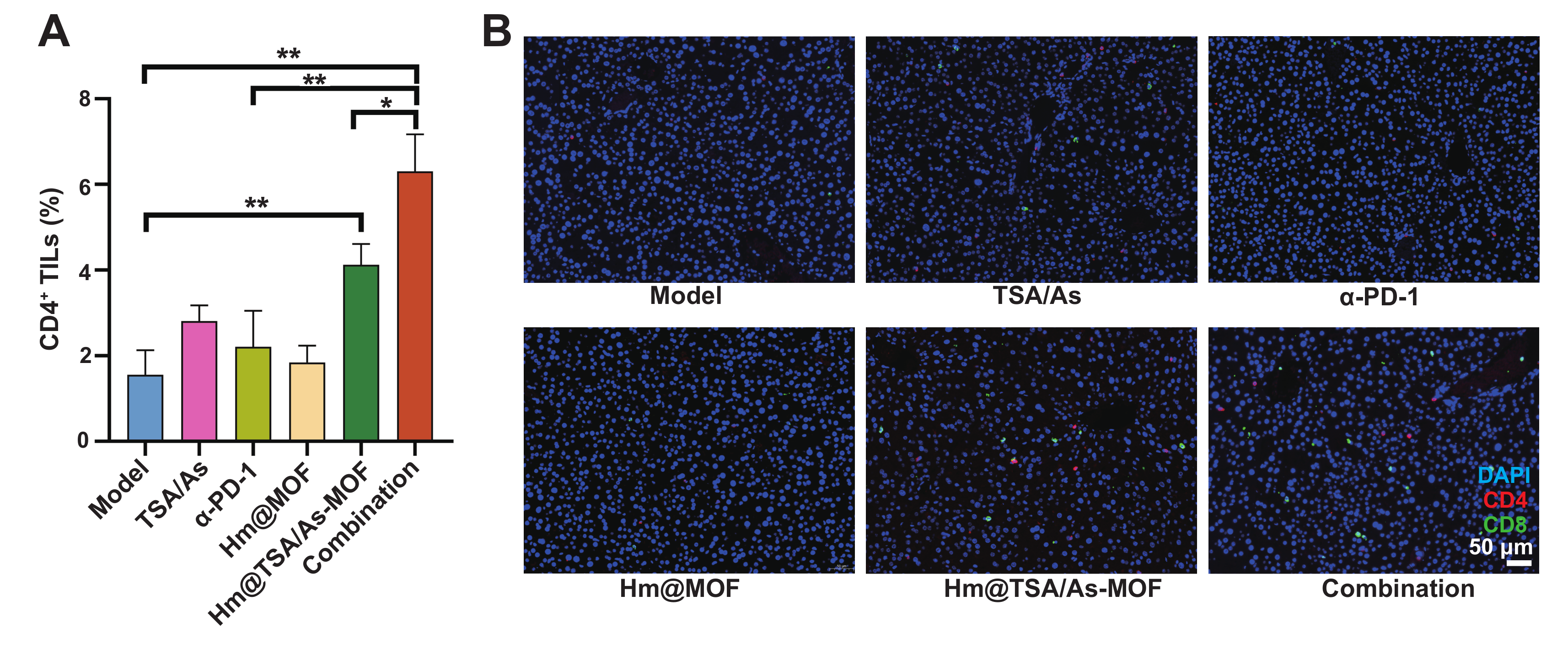


**Figure S8**. Levels of CD4^+^ (A) were demonstrated by flow cytometry (n=3). CD4^+^ and CD8^+^ TILs (B) were labeled on tumor tissue sections by fluorescent staining (scale bar = 50 μm). Data represent the means ± SD. (*, *p* < 0.05; **, *p* < 0.01; two-tailed Student’s t-test).
